# Health-related quality of life and its associated factors in patients with chronic obstructive pulmonary disease

**DOI:** 10.1371/journal.pone.0293342

**Published:** 2023-10-26

**Authors:** Anan S. Jarab, Walid Al-Qerem, Karem H. Alzoubi, Shrouq Abu Heshmeh, Tareq L. Mukattash, Abdallah Y. Naser, Yazid N. Al Hamarneh

**Affiliations:** 1 Department of Clinical Pharmacy, Faculty of Pharmacy, Jordan University of Science and Technology, Irbid, Jordan; 2 College of Pharmacy, Al Ain University, Abu Dhabi, United Arab Emirates; 3 Department of Pharmacy, Faculty of Pharmacy, Al-Zaytoonah University of Jordan, Amman, Jordan; 4 Department of Pharmacy Practice and Pharmacotherapeutics, College of Pharmacy, University of Sharjah, Sharjah, United Arab Emirates; 5 Faculty of Pharmacy, Jordan University of Science and Technology, Irbid, Jordan; 6 Department of Applied Pharmaceutical Sciences and Clinical Pharmacy, Faculty of Pharmacy, Isra University, Amman, Jordan; 7 Department of Pharmacology, Faculty of Medicine and Dentistry, University of Alberta, Edmonton, Canada; Nova Southeastern University / Yarmouk University, UNITED STATES

## Abstract

**Objective:**

The present study aimed to evaluate HRQOL and to explore the factors associated with poor HRQOL among patients with COPD.

**Methods:**

In the present cross-sectional study, the validated *St George’s Respiratory Questionnaire for COPD patients* (SGRQ-C) was used to evaluate HRQOL among 702 patients with COPD at two major hospitals in Jordan in the period between January and April 2022. Quantile regression analysis was used to explore the factors associated with HRQOL among the study participants.

**Results:**

According to SGRQ-C, the HRQOL of the study participants was greatly impaired with a total SGRQ of 55.2 (34–67.8). The highest impairment in the HRQOL was in the impact domain with a median of 58.7 (29–76.3). Increased number of prescribed medications (β = 1.157, P<0.01), older age (β = 0.487, P<0.001), male gender (β = 5.364, P<0.01), low education level (β = 9.313, P<0.001), low and moderate average income (β = 6.440, P<0.05, and β = 6.997, P<0.01, respectively) were associated with poorer HRQOL. On the other hand, being married (β = -17.122, P<0.001), living in rural area (β = -6.994, P<0.01), non-use of steroids inhalers (β = -3.859, P<0.05), not receiving long acting muscarinic antagonists (LAMA) (β = -9.269, P<0.001), not receiving LABA (β = -8.243, P<0.001) and being adherent to the prescribed medications (β = -6.016, P<0.001) were associated with improved HRQOL. Furthermore, lower disease severity (stage A, B, and C) (β = -23.252, -10.389, and -9.696 respectively, P<0.001), and the absence of comorbidities (β = -14.303, P<0.001) were associated with better HRQOL.

**Conclusions:**

In order to maximize HRQOL in patients with COPD, future COPD management interventions should adopt a multidisciplinary approach involving different healthcare providers, which aims to provide patient-centered care, implement personalized interventions, and improve medication adherence, particularly for patients who are elderly, males, have low socioeconomic status, receive multiple medications and have multiple comorbid diseases.

## Introduction

Chronic obstructive pulmonary disease (COPD) is a slow progressive disease characterized by pulmonary malfunction and airway obstruction, with cough, shortness of breath, and sputum production being the most common symptoms [1, 2]. COPD comprises two primary forms: chronic bronchitis characterized by a persistent cough with mucus, and emphysema, which involves gradual lung damage over time [3]. It is a chronic, incurable respiratory condition that affects around 13.1% of the global population [4]. Among adult Jordanians, the estimated prevalence of COPD was 5.4%, whereas it reached 11% in current male smokers [5, 6]. In 2019, the World Health Organization rated COPD as the third leading cause of death, accounting for 6% of the world’s total deaths [7]. Uncontrolled COPD has been associated with reduced health-related quality of life (HRQOL), higher work productivity and activity impairment, and greater use of healthcare resources [8, 9].

HRQOL is a multidimensional concept that captures information on patients’ physical and mental health state in order to assess the influence of the health status on the quality of life [10]. As is the case with other chronic diseases [11], several studies report impaired HRQOL in patients with COPD [12–17]. In Jordan, low HRQOL was observed among patients with COPD based on the St. George Respiratory Questionnaire (SGRQ) [18, 19]. In addition, it was found that nearly half of adults with COPD had poor health status when compared with only 15% of those without the disease [20]. Improving HRQOL has been recognized as one of the primary goals in the management of COPD by the Global Initiative for Chronic Obstructive Lung Disease (GOLD) guidelines [2]. Nevertheless, the variables that are associated with poor HRQOL in COPD patients have to be revealed in order to build appropriate interventions that would help improve quality of life in these patients.

The literature reported inconsistent findings regarding the variables that negatively impact HRQOL in patients with COPD. Earlier studies reported that worse HRQOL was found in patients with COPD who had frequent exacerbations or have multiple comorbidities [14, 17, 21]. Other studies found significant association between disease severity and poor HRQOL in patients with COPD [8, 12–14]. Smoking was also recognized as determinant for the impaired HRQOL in COPD patients [15, 21]. Furthermore, the advanced age was associated with reduced HRQOL in patients with COPD in multiple previous studies [15, 17]. Nevertheless, the onset of COPD at an earlier age was found to have a greater deterioration on HRQOL due to the early onset of symptoms and complications related to this disease [13]. Aside from physical health decline, people with COPD may feel anxiety and depression owing to difficulty to complete everyday activities, which can have a significant impact on HRQOL [22]. Exploring the factors associated with poor HRQOL in COPD patients is deemed necessary in order to come up with strategies that would help enhancing HRQOL and health outcomes in this group of patients. Therefore, the aim of this study was to assess mental wellbeing measurements such as anxiety and depression, HRQOL and to investigate the factors associated with poor HRQOL among patients with COPD.

## Methods

### Study design and subjects

The present cross-sectional study was conducted on patients with COPD who attended the outpatient respiratory clinic at King Abdullah University Hospital (KAUH) and the Royal Medical Services (RMS) in the period from January through April, 2022. Participants who were 18 years or older and had a confirmed diagnosis of COPD for at least 6 months were eligible to participate in the study. Patients with congestive heart failure, cognitive impairment, sever disease, or other pulmonary problems were excluded from the study. During the outpatient respiratory clinic visit, patients who met the inclusion criteria were invited by the researcher (SA) to participate in the study, and were informed that participation is voluntary and they have the right to refuse to participate or withdraw from the study at any time. Patients who agreed to participate were asked to sign a written informed consent. In a separate room at the clinic, the survey was administered face to face to and self-completed by the participants using an electronic device, Information about age, gender, education level, income, smoking status, marital status, living conditions, living area, and having pets were collected using a custom-designed questionnaire. Patient interviews and medical files were used to collect information about the disease and medication-related factors including disease duration, reported disease severity, comorbidities such as hypertension, heart diseases, diabetes, number of COPD medications, number of total medications, receiving inhaled corticosteroids, oral steroids, long-acting B_2_ agonist (LABA), (LAMA), short-acting B_2_ agonist (SABA), use of oxygen cylinders, having concerns about the side effects of COPD medication, the appropriate use of inhalers, patients’ evaluation of the drug effectiveness, anxiety level, depression level, medication adherence, and GOLD disease severity. In addition to evaluating medication adherence using the 4-item Medication adherence scale [23], the patients were asked to describe how they use the inhaler device, and the responses were assessed using a validated checklist adapted from the German Respiratory League’s recommendations [24].

### Study instruments

An expert panel of different background including two pulmonologists and two professors of public health examined the study questionnaire for face and content validity. The survey was also piloted on ten COPD patients to investigate the clarity of the questions, relevance of items, and time for completion. Data obtained from the pilot test was not included in the final data analysis.

### St George’s Respiratory Questionnaire for COPD patients (SGRQ-C)

The SGRQ-C is a shorter version derived from the original SGRQ, which was specifically developed and validated for the measurement of health-related quality of life (HRQOL) in patients with asthma or COPD. Since the SGRQ-C was developed using COPD data, it is therefore only valid for use in this disease [25]. The 14-item questionnaire included two parts with seven questions for each one. The first part was designed to assess the frequency of respiratory symptoms (symptoms component), while the second part addressed the patient’s current state in terms of disturbances to daily physical activity (activity component) and disturbances to psycho-social function (impacts component). The total and the three components’ scores were calculated. The scores ranged from 0 to 100%, with higher values indicating poorer health status [25]. The validated Arabic version of SGRQ-C was used in this study [26, 27].

### Hospital Anxiety and Depression scale (HADS)

The HADS was created in 1983 as a reliable and valid tool for detecting depression and anxiety in hospital outpatient settings [28]. The 14-item test yields two scores: one for anxiety and the other for depression. Each item is graded on a scale of 0 to 3 (no impairment) with a maximum score of 21 for anxiety or depression. Patients with a score of 11 or higher on any scale were considered to have a definitive case of anxiety, depression, or both [29]. The validated Arabic version of HADS was used in this study [30].

### Sample size calculation

The following equation was used to estimate the minimum sample size required to determine the variables associated with HRQOL: N>50+8p [31], where p is the number of predictors. As the current study evaluated 16 variables, the required sample size was 175.

### Statistical analysis

Data analysis was conducted using SPSS. Categorical variables were presented as frequencies and percentages. Q-Q plots indicated that the data was not normally and therefore it was presented as medians and interquartile range (IQR) and analyzed using nonparametric tests. Quantile regression was used to identify the variables associated with HRQOL among the study patients. The independent variables included in the model were age, sex, educational level, marital status, residency, use of inhaled steroids, LAMA, SABA and LABA, number of medications, adherence level, smoking status, income level, disease severity, the presence of other comorbidities and disease duration. A P-value of < 0.05 was considered statistically significant.

### Ethics approval

The study has been conducted in accordance with The Code of Ethics of the World Medical Association (Declaration of Helsinki) for experiments involving human subjects. The study received ethical approval from the Institutional Review Board at Jordan University of Science and Technology, Irbid, Jordan and the Royal Medical Services, Amman, Jordan (Ref. # 29/139/2021).

## Results

A total of 702 patients participated in the study. The median age was 68 years (58–77). The majority of the participants were males (78.6%), married (91.5%), smokers (58%), not living alone (93%), and lived in urban area (86.9%), while nearly half of them had low income (47%). More details about the socio-demographic characteristics of the study sample are presented in Table 1.

**Table 1:** Socio-demographic characteristics of the study participants (n = 702).

Variable		Median (25^th^-75^th^ quartiles)	Frequency (percentage %)
Age (years)		68 (58–77)	
Gender	Male		552 (78.6)
	Female		150 (21.4)
Educational level	Low		252 (35.9)
	Moderate		228 (32.5)
	High		222 (31.6)
Average income	less than 600 JD		330 (47.0)
	600–1000 JD		290 (41.3)
	more than 1000 JD		82 (11.7)
Material status	Not married		0 (0)
	Married		642 (91.5)
	Other		60 (8.5)
Smoking	Former smoker		295 (42.0)
	Smoker		407 (58.0)
Living conditions	Alone		49 (7.0)
	Not alone		653 (93.0)
Living area	Rural area		92 (13.1)
	Urban area		610 (86.9)
Having pets	No		636 (90.6)
	Yes		66 (9.4)

JD: Jordanian dinar.

As shown in Table 2, the majority of the participants had hypertension (72.6%), diabetes (50.4%), received inhaled corticosteroids (68.4%), rated medications’ effectiveness good (59%) and were non-adherent to COPD medications (67.9%). Although most of the participants were not suffering from anxiety (59%), 54.7% of them were depressed. According to the GOLD disease severity, most of the participants were in group B (40.2%). The median of the inhaler score was estimated as 91.4% (74.4–100), indicating good knowledge about the appropriate use of inhalers among the study participants. According to SGRQ-C, the HRQOL of the study participants was greatly impaired with a total SGRQ-C of 55.2 (34–67.8). The highest impairment in the HRQOL was in the impact domain with a median of 58.7 (29–76.3).

**Table 2:** Medical characteristics of the study participants (n = 702).

Variable			Median (25th-75th quartiles)	Frequency (percentage%)
Disease duration			4 (2–5)	
Comorbidities	Hypertension	No		192 (27.4)
		Yes		510 (72.6)
	Heart diseases	No		366 (52.1)
		Yes		336 (47.9)
	Diabetes	No		348 (49.6)
		Yes		354 (50.4)
Number of COPD medications			2 (2–2)	
Number of total medications			5 (3–6)	
Inhaled corticosteroids		No		222 (31.6)
		Yes		480 (68.4)
Oral steroids		No		678 (96.6)
		Yes		24 (3.4)
LABA		No		272 (38.7)
		Yes		430 (61.3)
LAMA		No		396 (56.4)
		Yes		306 (43.6)
SABA		No		564 (80.3)
		Yes		138 (19.7)
Use of oxygen cylinders		No		546 (77.8)
		Yes		156 (22.2)
having concerns about the side effects of COPD medication		No		594 (84.6)
		Yes		108 (15.4)
Inhaler score percent			91.4 (74.4–100)	
Perceived medication effectiveness		Not good		54 (7.7)
		Good		414 (59.0)
		Excellent		234 (33.3)
Anxiety level		Normal		414 (59.0)
		Abnormal		288 (41.0)
Depression level		Normal		318 (45.3)
		Abnormal		384 (54.7)
Medication adherence		Non-adherent		477 (67.9)
		Adherent		225 (32.1)
GOLD disease severity group		A		114 (16.2)
		B		282 (40.2)
		C		108 (15.4)
		D		198 (28.2)
SGRQ-C scores		SGRQ symptoms score	45.2 (32.8–59.8)	
		SGRQ activity score	51.7 (22.5–67.6)	
		SGRQ impact score	58.7 (29–76.3)	
		SGRQ total score	55.2 (34–67.8)	

COPD: chronic obstructive pulmonary disease, LABA: long-acting B_2_ agonist, LAMA: long-acting muscarinic antagonist, SABA: short-acting B_2_ agonist. SGRQ: St George’s Respiratory Questionnaire.

As presented in Table 3, Quantile regression results showed that higher number of prescribed medications (β = 1.157, P<0.01), older age (β = 0.487, P<0.001), male gender (β = 5.364, P<0.01), low education level (β = 9.313, P<0.001), low and moderate average income (β = 6.440, P<0.05, and β = 6.997, P<0.01, respectively) were associated with poorer HRQOL. On the other hand, being married (β = -17.122, P<0.001), living in rural area (β = -6.994, P<0.01), non-use of steroids inhalers (β = -3.859, P<0.05), not receiving LAMA (β = -9.269, P<0.001), not receiving LABA (β = -8.243, P<0.001), being adherent to medications (B = -6.016, P<0.001), lower disease severity (stage A, B, and C) (β = -23.252, -10.389, and -9.696 respectively, P<0.001), and the absence of comorbidities (β = -14.303, P<0.001) were associated with better HRQOL scores.

**Table 3:** Multivariate analysis of the factors associated with HRQOL.

Parameter		Quantile regression coefficient	P	95% Confidence Interval	
				Lower	Upper
How many medications do you generally take?		1.157	0.008**	0.310	2.004
Age		0.487	<0.001**	0.356	0.618
Disease duration		0.087	0.732	-0.413	0.587
Gender	Male	5.364	0.009**	1.365	9.363
	Female	Reference	.	.	.
Educational level	Low	9.313	<0.001**	5.165	13.460
	Moderate	-0.694	0.729	-4.626	3.238
	High	Reference	.	.	.
Material status	Married	-17.122	<0.001**	-22.639	-11.605
	Other	Reference	.	.	.
Living area	Rural area	-6.994	0.003**	-11.631	-2.358
	Urban area	Reference	.	.	.
Steroids inhaler use	No	-3.859	0.018*	-7.063	-0.656
	Yes	Reference	.	.	.
Receiving LAMA	No	-9.269	<0.001**	-12.540	-5.997
	Yes	Reference	.	.	.
Receiving SABA	No	-2.942	0.142	-6.873	0.989
	Yes	Reference	.	.	.
Adherence level	Adherent	-6.016	0.001**	-9.546	-2.487
	Non-adherent	Reference	.	.	.
Receiving LABA	No	-8.243	<0.001**	-11.441	-5.045
	Yes	Reference	.	.	.
Smoking	Former smoker	-1.423	0.363	-4.490	1.644
	Smoker	Reference	.	.	.
Average income	<600 JOD	6.440	0.014*	1.313	11.567
	600–1000 JOD	6.997	0.008**	1.802	12.192
	>1000 JOD	Reference	.	.	.
Disease severity	A	-23.252	<0.001**	-28.338	-18.167
	B	-10.389	<0.001**	-14.240	-6.537
	C	-9.696	<0.001**	-14.492	-4.901
	D	Reference	.	.	.
Comorbidities	No	-14.303	<0.001**	-19.757	-8.849
	Yes	Reference			

* Significant at P<0.05,

** Significant at P<0.01.

## Discussion

The fact that COPD patients’ HRQOL is markedly reduced at all levels of disease severity, even in those with mild COPD [14, 32, 33], emphasizes the significance of identifying the determinants of these patients’ poor HRQOL, which was the main goal of the current study. The results of this study should serve as a blueprint for developing future pharmaceutical care programs that improve COPD patients’ HRQOL and maximize the health outcomes. Jordan is widely acclaimed for its top-notch healthcare services, making it a prominent destination for medical tourism in the Middle East and North Africa region [34]. However, in recent decades, the healthcare system in Jordan has encountered several challenges that may have impacted the quality of life for patients in the country. These challenges include the escalating demand for health services due to population growth, a shift in disease patterns with a higher prevalence of non-communicable diseases and a decrease in communicable diseases, the presence of refugees, an expected increase in the proportion of both young and elderly populations, and the rising healthcare costs amidst the prevailing economic situation [35].

In this study, COPD patients were found to have substantially impaired HRQOL, which was consistent with the results reported in earlier studies [21, 36, 37], but worse than those found in other studies [14, 38–40]. Moreover, a study conducted in four Gulf Council Cooperation countries revealed a significant compromise in the HRQOL among patients living with COPD [41]. Another study was conducted to evaluate HRQOL in three chronic diseases including COPD, heart failure, and chronic kidney disease in Oman revealed that COPD patients exhibited the lowest HRQOL scores [42].

Consistent with previous research findings [43–47], this study found a strong relationship between the high number of prescribed medications and poor HRQOL. Older participants have also demonstrated worse HRQOL, when compared to younger ones in our investigation. Previous studies reported similar findings [15, 48]. Age was reported as one of the significant predictors affecting HRQOL in patients with COPD in an integrative review [49]. Furthermore, the presence of comorbid diseases along with COPD significantly reduced HRQOL in COPD patients in this study, which was in line with the findings reported in earlier studies conducted in Hungary [21], India [48], and Nepal [50]. The association between these aforementioned variables and poor HRQOL can be explained by the fact that as patients age, they may develop additional complications and diseases that require more medications to manage them, leading to medication non-adherence and poor HRQOL. A systematic review reported medication non-adherence rates ranging from 22% to 93% in patients with COPD [51], which increased the risk for COPD exacerbations and poor HRQOL [52, 53]. Consistent with earlier study finding [54], medication non-adherence was associated with poor HRQOL. Previous interventional trials reported that personalized pharmaceutical care interventions improved medication adherence, decreased hospitalization, and enhanced HRQOL in COPD patients [55, 56], highlighting the importance of implementing these interventions through pharmacist-led programs to enhance medication adherence and HRQOL in COPD patients, specifically targeting older patients, those receiving higher number of medications and/or having comorbidities.

Several socio-demographic variables were associated with reduced HRQOL in this study, such as male gender, low education level, low-to-moderate income, living in urban areas, and not being married. Previous literature has observed a strong relationship between low socioeconomic status and low HRQOL [57–61]. Due to the multifactorial nature of this association and the potential role that social, economic, environmental, and behavioral factors may play in it, it is important to consider these factors when creating effective treatment plans for COPD patients. It is also important to be aware of the negative effects that these factors may have on the HRQOL of COPD patients, and to create appropriate strategies accordingly.

In this study, patients with COPD who were not receiving steroids inhalers, LAMA, or LABA had significantly better HRQOL. This finding could be attributed to the bothersome side effects of these medications, such as oropharyngeal candidiasis, skin thinning, pneumonia, and cataract with ICS [62, 63], dry mouth, constipation and urinary retention with LAMA [64], and tremor, palpitations, and hypokalemia with LABA [65], which may affect HRQOL. It is important to note that prior randomized controlled trials reported that ICS in combination with LABA has been associated with superior results in terms of HRQOL when compared with the use of LABA or LAMA alone in patients with COPD [66–68]. Additionally, triple therapy with ICS, LABA, and LAMA was proven to improve HRQOL and lessen dyspnea to a greater extent than LABA/LAMA or ICS/LABA therapy [69]. Thus, when examining the impact of these medications on the HRQOL of COPD patients, the results may vary depending on whether the medication’s effect was investigated individually, or in combination with other medications, which warrants further investigation in this area to reduce this inconsistency and reveal the truth.

In the current study, HRQOL was considerably impacted by the severity of COPD as determined by the new GOLD criteria, with patients in grade D exhibiting significantly worse HRQOL than those in lower severity grades. Similar results were reported in previous studies conducted in Thailand [70, 71], India [13, 15, 48], Jordan [49], and Egypt [12, 17]. Based on these findings, future efforts should concentrate on targeted actions and plans to improve HRQOL, especially for patients classified in grade D, as these people have significantly worse HRQOL than those with lower severity grades. This suggests a need for tailored assistance and comprehensive management strategies that would improve COPD control and lessen its severity, and thereby improve HRQOL and health outcomes for these patients.

Contrary to the results reported in previous studies [72–74], the present study found no significant association between smoking and reduced HRQOL. Several factors may contribute to this discrepancy such as variation in patients’ characteristics, study design, instruments used, and smoking behavior and habits. It is important to acknowledge the limitations that might affect the interpretation of our findings. The cross-sectional design used in this study limits the ability to establish causal relationships and measure the longitudinal outcomes. The study was conducted in only two hospital sites and did not include all the Jordanian provinces, which might limit the generalizability of the study findings. Furthermore, the convenient sampling technique used in this study might cause selection bias. Moreover, the self-report method used in the study survey may have exposed the participants’ responses to social-desirability bias.

## Conclusions

The current study showed markedly impaired HRQOL among patients with COPD in Jordan. Adopting a multidisciplinary approach that involves various healthcare professionals can optimize COPD management and ultimately lead to better HRQOL outcomes. Physicians can incorporate routine HRQOL measurement before prescribing treatments, facilitating more patient-centered care and informed decision-making. Additionally, pharmacists can play a crucial role in monitoring patients’ medication adherence over time, identifying barriers and concerns that may hinder patients’ adherence to the prescribed regimens. This collaborative effort, which aims to enhance the overall well-being and health outcomes among COPD patients, should focus on elderly patients, males, patients with low socioeconomic status and those who receive multiple medications and have multiple comorbid diseases.

## Supporting information

S1 Questionnaire Inclusivity in global research. (DOCX)

S1 Data (PDF)
