# Supplementary material for: Health-related quality of life and its associated factors in patients with chronic obstructive pulmonary disease
Source: PLoS One. 2023 Oct 26;18(10):e0293342. doi: 10.1371/journal.pone.0293342 (PMC10602230; doi:10.1371/journal.pone.0293342)
Supplement: S1 Data — (PDF) [file pone.0293342.s002.pdf]

Quantile Regression

Model Quality (q=0.5)[a,b,c]

Pseudo R Squared	.336
Mean Absolute Error (MAE)	12.6074

a. Dependent Variable: SGRQ_Perc

b. Model: (Intercept), How many medications do you generally take?, Age, Disease duration, Gender , Educational level , Material status , Living in , Steroids inhaler, LAMA, SABA, Adherance_level, LABA, Smoking , Average income, Disease severity , binary_chronic

c. Method: Simplex algorithm

Parameter Estimates (q=0.5)[a,b]

Parameter	Coefficient	Std. Error	t	df	Sig.	95% Confidence Interval Lower Bound	95% Confidence Interval Upper Bound
(Intercept)	39.608	7.0533	5.616	681	.000	25.760	53.457
How many medications do you generally take?	1.157	.4315	2.682	681	.008	.310	2.004
Age	.487	.0666	7.308	681	.000	.356	.618
Disease duration	.087	.2546	.342	681	.732	-.413	.587
[Gender =0]	5.364	2.0366	2.634	681	.009	1.365	9.363
[Gender =1]	0[c]	.	.	.	.	.	.
[Educational level =1]	9.313	2.1125	4.408	681	.000	5.165	13.460
[Educational level =2]	-.694	2.0025	-.347	681	.729	-4.626	3.238
[Educational level =3]	0[c]	.	.	.	.	.	.
[Material status =2]	-17.122	2.8098	-6.094	681	.000	-22.639	-11.605
[Material status =3]	0[c]	.	.	.	.	.	.
[Living in =1]	-6.994	2.3615	-2.962	681	.003	-11.631	-2.358
[Living in =2]	0[c]	.	.	.	.	.	.
[Steroids inhaler=.00]	-3.859	1.6316	-2.365	681	.018	-7.063	-.656
[Steroids inhaler=1.00]	0[c]	.	.	.	.	.	.
[LAMA=.00]	-9.269	1.6660	-5.563	681	.000	-12.540	-5.997
[LAMA=1.00]	0[c]	.	.	.	.	.	.
[SABA=.00]	-2.942	2.0021	-1.469	681	.142	-6.873	.989
[SABA=1.00]	0[c]	.	.	.	.	.	.
[Adherance_level=.00]	-6.016	1.7976	-3.347	681	.001	-9.546	-2.487
[Adherance_level=1.00]	0[c]	.	.	.	.	.	.
[LABA=.00]	-8.243	1.6287	-5.061	681	.000	-11.441	-5.045
[LABA=1.00]	0[c]	.	.	.	.	.	.
[Smoking =2]	-1.423	1.5620	-.911	681	.363	-4.490	1.644
[Smoking =3]	0[c]	.	.	.	.	.	.
[Average income=1]	6.440	2.6113	2.466	681	.014	1.313	11.567
[Average income=2]	6.997	2.6461	2.644	681	.008	1.802	12.192
[Average income=3]	0[c]	.	.	.	.	.	.
[Disease severity =1]	-23.252	2.5899	-8.978	681	.000	-28.338	-18.167

Parameter Estimates (q=0.5)[a,b]

Parameter	Coefficient	Std. Error	t	df	Sig.	95% Confidence Interval Lower Bound	95% Confidence Interval Upper Bound
[Disease severity =2]	-10.389	1.9616	-5.296	681	.000	-14.240	-6.537
[Disease severity =3]	-9.696	2.4424	-3.970	681	.000	-14.492	-4.901
[Disease severity =4]	0[c]	.	.	.	.	.	.
[binary_chronic=.00]	-14.303	2.7779	-5.149	681	.000	-19.757	-8.849
[binary_chronic=1.00]	0[c]	.	.	.	.	.	.

a. Dependent Variable: SGRQ_Perc

b. Model: (Intercept), How many medications do you generally take?, Age, Disease duration, Gender , Educational level , Material status , Living in , Steroids inhaler, LAMA, SABA, Adherance_level, LABA, Smoking , Average income, Disease severity , binary_chronic

c. Set to zero because this parameter is redundant.

Prediction: How many medications do you generally take?

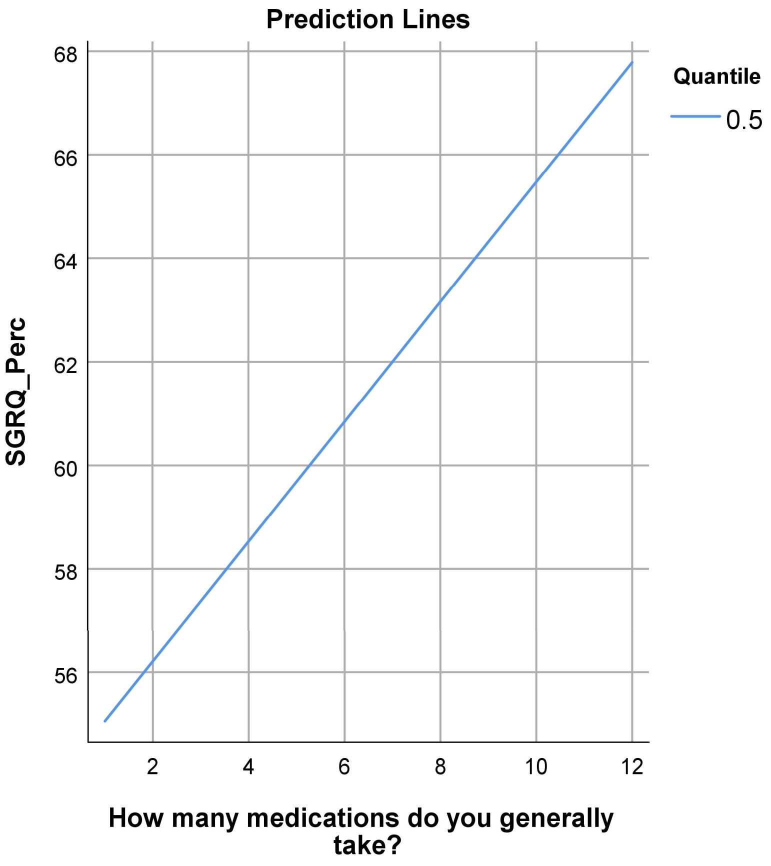

Prediction: Age

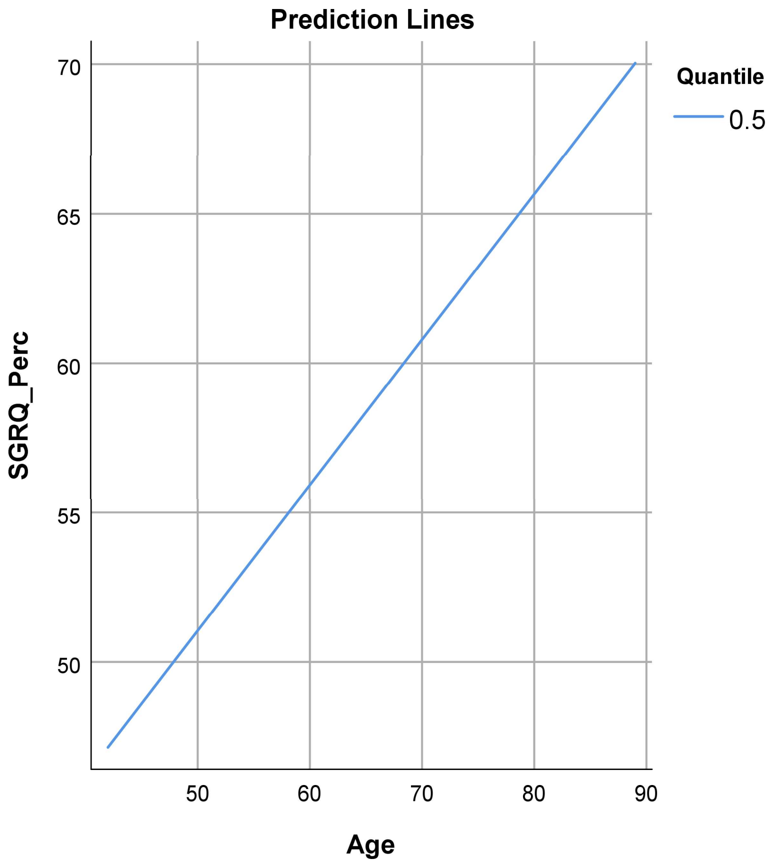

Prediction: Disease duration

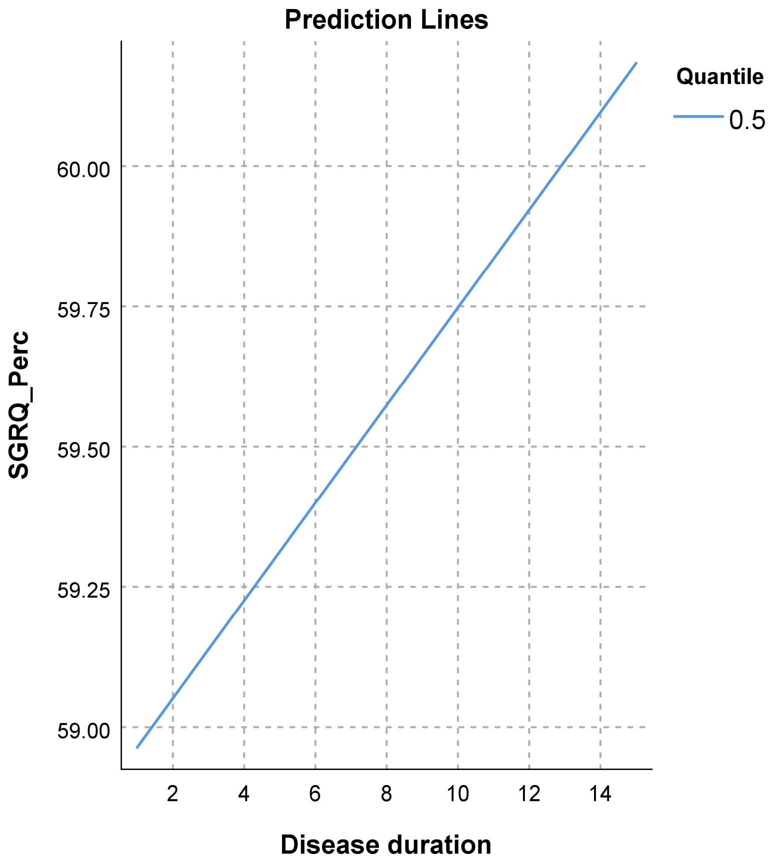

Prediction: Gender

Prediction Table[a,b,c]

Gender	q=0.5
Male	59.238
Female	53.874

a. Dependent Variable: SGRQ_Perc

b. Model: (Intercept), How many medications do you generally take?, Age, Disease duration, Gender , Educational level , Material status , Living in , Steroids inhaler, LAMA, SABA, Adherance_level, LABA, Smoking , Average income, Disease severity , binary_chronic

c. Predictors in the model are evaluated at Educational level =Low,Material status =Married,Living in =Urban area,Steroids inhaler=Yes,LAMA=No,SABA=No,Adherance_level=non adhered,LABA=Yes, Smoking =Smoker,Average income=less than 600,Disease severity =B,binary_chronic=Yes,How many medications do you generally take?=4.62,Age=66.8262,Disease duration=4.1496

Prediction: Educational level

Prediction Table[a,b,c]

Educational level	q=0.5
Low	59.238
Moderate	49.232
High	49.926

a. Dependent Variable: SGRQ_Perc

b. Model: (Intercept), How many medications do you generally take?, Age, Disease duration, Gender , Educational level , Material status , Living in , Steroids inhaler, LAMA, SABA, Adherance_level, LABA, Smoking , Average income, Disease severity , binary_chronic

c. Predictors in the model are evaluated at Gender =Male,Material status =Married,Living in =Urban area, Steroids inhaler=Yes,LAMA=No,SABA=No,Adherance_level=non adhered,LABA=Yes,Smoking =Smoker,Average income=less than 600,Disease severity =B,binary_chronic=Yes,How many medications do you generally take?=4.62,Age=66.8262,Disease duration=4.1496

Prediction: Material status

Prediction Table[a,b,c]

Material status	q=0.5
Married	59.238
Other	76.361

a. Dependent Variable: SGRQ_Perc

b. Model: (Intercept), How many medications do you generally take?, Age, Disease duration, Gender , Educational level , Material status , Living in , Steroids inhaler, LAMA, SABA, Adherance_level, LABA, Smoking , Average income, Disease severity , binary_chronic

c. Predictors in the model are evaluated at Gender =Male,Educational level =Low,Living in =Urban area, Steroids inhaler=Yes,LAMA=No,SABA=No,Adherance_level=non adhered,LABA=Yes,Smoking =Smoker,Average income=less than 600,Disease severity =B,binary_chronic=Yes,How many medications do you generally take?=4.62,Age=66.8262,Disease duration=4.1496

Prediction: Living in

Prediction Table[a,b,c]

Living in	q=0.5
Rural area	52.244
Urban area	59.238

a. Dependent Variable: SGRQ_Perc

b. Model: (Intercept), How many medications do you generally take?, Age, Disease duration, Gender , Educational level , Material status , Living in , Steroids inhaler, LAMA, SABA, Adherance_level, LABA, Smoking , Average income, Disease severity , binary_chronic

c. Predictors in the model are evaluated at Gender =Male,Educational level =Low,Material status =Married, Steroids inhaler=Yes,LAMA=No,SABA=No,Adherance_level=non adhered,LABA=Yes,Smoking =Smoker,Average income=less than 600,Disease severity =B,binary_chronic=Yes,How many medications do you generally take?=4.62,Age=66.8262,Disease duration=4.1496

Prediction: Steroids inhaler

Prediction Table[a,b,c]

Steroids inhaler	q=0.5
No	55.379
Yes	59.238

a. Dependent Variable: SGRQ_Perc

b. Model: (Intercept), How many medications do you generally take?, Age, Disease duration, Gender , Educational level , Material status , Living in , Steroids inhaler, LAMA, SABA, Adherance_level, LABA, Smoking , Average income, Disease severity , binary_chronic

c. Predictors in the model are evaluated at Gender =Male,Educational level =Low,Material status =Married, Living in =Urban area,LAMA=No,SABA=No,Adherance_level=non adhered,LABA=Yes,Smoking =Smoker,Average income=less than 600,Disease severity =B,binary_chronic=Yes,How many medications do you generally take?=4.62,Age=66.8262,Disease duration=4.1496

Prediction: LAMA

Prediction Table[a,b,c]

LAMA	q=0.5
No	59.238
Yes	68.507

a. Dependent Variable: SGRQ_Perc

b. Model: (Intercept), How many medications do you generally take?, Age, Disease duration, Gender , Educational level , Material status , Living in , Steroids inhaler, LAMA, SABA, Adherance_level, LABA, Smoking , Average income, Disease severity , binary_chronic

c. Predictors in the model are evaluated at Gender =Male,Educational level =Low,Material status =Married, Living in =Urban area,Steroids inhaler=Yes,SABA=No,Adherance_level=non adhered,LABA=Yes, Smoking =Smoker,Average income=less than 600,Disease severity =B,binary_chronic=Yes,How many medications do you generally take?=4.62,Age=66.8262,Disease duration=4.1496

Prediction: SABA

Prediction Table[a,b,c]

SABA	q=0.5
No	59.238
Yes	62.180

a. Dependent Variable: SGRQ_Perc

b. Model: (Intercept), How many medications do you generally take?, Age, Disease duration, Gender , Educational level , Material status , Living in , Steroids inhaler, LAMA, SABA, Adherance_level, LABA, Smoking , Average income, Disease severity , binary_chronic

c. Predictors in the model are evaluated at Gender =Male,Educational level =Low,Material status =Married, Living in =Urban area,Steroids inhaler=Yes,LAMA=No,Adherance_level=non adhered,LABA=Yes, Smoking =Smoker,Average income=less than 600,Disease severity =B,binary_chronic=Yes,How many medications do you generally take?=4.62,Age=66.8262,Disease duration=4.1496

Prediction: Adherance_level

Prediction Table[a,b,c]

Adherance_level	q=0.5
adhered	53.222
non adhered	59.238

a. Dependent Variable: SGRQ_Perc

b. Model: (Intercept), How many medications do you generally take?, Age, Disease duration, Gender , Educational level , Material status , Living in , Steroids inhaler, LAMA, SABA, Adherance_level, LABA, Smoking , Average income, Disease severity , binary_chronic

c. Predictors in the model are evaluated at Gender =Male,Educational level =Low,Material status =Married, Living in =Urban area,Steroids inhaler=Yes,LAMA=No,SABA=No,LABA=Yes,Smoking =Smoker,Average income=less than 600,Disease severity =B,binary_chronic=Yes,How many medications do you generally take?=4.62,Age=66.8262,Disease duration=4.1496

Prediction: LABA

Prediction Table[a,b,c]

LABA	q=0.5
No	50.996
Yes	59.238

a. Dependent Variable: SGRQ_Perc

b. Model: (Intercept), How many medications do you generally take?, Age, Disease duration, Gender , Educational level , Material status , Living in , Steroids inhaler, LAMA, SABA, Adherance_level, LABA, Smoking , Average income, Disease severity , binary_chronic

c. Predictors in the model are evaluated at Gender =Male,Educational level =Low,Material status =Married, Living in =Urban area,Steroids inhaler=Yes,LAMA=No,SABA=No,Adherance_level=non adhered, Smoking =Smoker,Average income=less than 600,Disease severity =B,binary_chronic=Yes,How many medications do you generally take?=4.62,Age=66.8262,Disease duration=4.1496

Prediction: Smoking

Prediction Table[a,b,c]

Smoking	q=0.5
Former smoker	57.816
Smoker	59.238

a. Dependent Variable: SGRQ_Perc

b. Model: (Intercept), How many medications do you generally take?, Age, Disease duration, Gender , Educational level , Material status , Living in , Steroids inhaler, LAMA, SABA, Adherance_level, LABA, Smoking , Average income, Disease severity , binary_chronic

c. Predictors in the model are evaluated at Gender =Male,Educational level =Low,Material status =Married, Living in =Urban area,Steroids inhaler=Yes,LAMA=No,SABA=No,Adherance_level=non adhered, LABA=Yes,Average income=less than 600,Disease severity =B,binary_chronic=Yes,How many medications do you generally take?=4.62,Age=66.8262,Disease duration=4.1496

Prediction: Average income

Prediction Table[a,b,c]

Average income	q=0.5
less than 600	59.238
600-1000	59.795
more than 1000	52.798

a. Dependent Variable: SGRQ_Perc

b. Model: (Intercept), How many medications do you generally take?, Age, Disease duration, Gender , Educational level , Material status , Living in , Steroids inhaler, LAMA, SABA, Adherance_level, LABA, Smoking , Average income, Disease severity , binary_chronic

c. Predictors in the model are evaluated at Gender =Male,Educational level =Low,Material status =Married, Living in =Urban area,Steroids inhaler=Yes,LAMA=No,SABA=No,Adherance_level=non adhered, LABA=Yes,Smoking =Smoker,Disease severity =B,binary_chronic=Yes,How many medications do you generally take?=4.62,Age=66.8262,Disease duration=4.1496

Prediction: Disease severity

Prediction Table[a,b,c]

Disease severity	q=0.5
A	46.375
B	59.238
C	59.931
D	69.627

a. Dependent Variable: SGRQ_Perc

b. Model: (Intercept), How many medications do you generally take?, Age, Disease duration, Gender , Educational level , Material status , Living in , Steroids inhaler, LAMA, SABA, Adherance_level, LABA, Smoking , Average income, Disease severity , binary_chronic

c. Predictors in the model are evaluated at Gender =Male,Educational level =Low,Material status =Married, Living in =Urban area,Steroids inhaler=Yes,LAMA=No,SABA=No,Adherance_level=non adhered, LABA=Yes,Smoking =Smoker,Average income=less than 600,binary_chronic=Yes,How many medications do you generally take?=4.62,Age=66.8262,Disease duration=4.1496

Prediction: binary_chronic

Prediction Table[a,b,c]

binary_chronic	q=0.5
No	44.935
Yes	59.238

a. Dependent Variable: SGRQ_Perc

b. Model: (Intercept), How many medications do you generally take?, Age, Disease duration, Gender , Educational level , Material status , Living in , Steroids inhaler, LAMA, SABA, Adherance_level, LABA, Smoking , Average income, Disease severity , binary_chronic

c. Predictors in the model are evaluated at Gender =Male,Educational level =Low,Material status =Married, Living in =Urban area,Steroids inhaler=Yes,LAMA=No,SABA=No,Adherance_level=non adhered, LABA=Yes,Smoking =Smoker,Average income=less than 600,Disease severity =B,How many medications do you generally take?=4.62,Age=66.8262,Disease duration=4.1496

```
QUANTILE REGRESSION SGRQ_Sympt_Perc BY Sex Educational_level Marital_Status Re
sidency
    Steroids_inhaler LAMA SABA Adherance_level LABA Smoking_status Income Dise
ase_severity
    binary_chronic WITH Number_of_medications_ingeneral Age Disease_duration
  /CRITERIA QUANTILE=0.5 METHOD=AUTO IID=TRUE BANDWIDTH=BOFINGER TOL=0.0000000
00001 CONV=0.000001
    MAXITER=2000 CILEVEL=95
  /MISSING CLASSMISSING=EXCLUDE
  /MODEL INTERCEPT=TRUE
  /PRINT PARAMETER
  /PLOT  PREDICTED_BY_OBSERVED=FALSE MAX_CATEGORIES=20
  /PREDICT_EFFECTS NUM_TOP_EFFECTS=20
  /SAVE  PRED.
```

Quantile Regression

Model Quality (q=0.5)[a,b,c]

Pseudo R Squared	.201
Mean Absolute Error (MAE)	13.4373

a. Dependent Variable: SGRQ_Sympt_Perc

b. Model: (Intercept), How many medications do you generally take?, Age, Disease duration, Gender , Educational level , Material status , Living in , Steroids inhaler, LAMA, SABA, Adherance_level, LABA, Smoking , Average income, Disease severity , binary_chronic

c. Method: Simplex algorithm

Parameter Estimates (q=0.5)[a,b]

Parameter	Coefficient	Std. Error	t	df	Sig.	95% Confidence Interval Lower Bound	95% Confidence Interval Upper Bound
(Intercept)	41.128	6.3899	6.436	681	.000	28.582	53.674
How many medications do you generally take?	.999	.3909	2.556	681	.011	.232	1.767
Age	.162	.0604	2.678	681	.008	.043	.280
Disease duration	.176	.2306	.764	681	.445	-.277	.629
[Gender =0]	1.630	1.8450	.883	681	.377	-1.993	5.253
[Gender =1]	0[c]	.	.	.	.	.	.
[Educational level =1]	1.838	1.9138	.961	681	.337	-1.919	5.596
[Educational level =2]	4.116	1.8141	2.269	681	.024	.554	7.678
[Educational level =3]	0[c]	.	.	.	.	.	.
[Material status =2]	-1.484	2.5455	-.583	681	.560	-6.482	3.514
[Material status =3]	0[c]	.	.	.	.	.	.
[Living in =1]	10.728	2.1394	5.014	681	.000	6.527	14.928
[Living in =2]	0[c]	.	.	.	.	.	.
[Steroids inhaler=.00]	2.995	1.4782	2.026	681	.043	.092	5.897
[Steroids inhaler=1.00]	0[c]	.	.	.	.	.	.
[LAMA=.00]	3.118	1.5093	2.066	681	.039	.154	6.081
[LAMA=1.00]	0[c]	.	.	.	.	.	.
[SABA=.00]	-1.192	1.8138	-.657	681	.511	-4.753	2.369
[SABA=1.00]	0[c]	.	.	.	.	.	.
[Adherance_level=.00]	-7.756	1.6285	-4.763	681	.000	-10.954	-4.559
[Adherance_level=1.00]	0[c]	.	.	.	.	.	.
[LABA=.00]	-7.028	1.4755	-4.763	681	.000	-9.925	-4.131
[LABA=1.00]	0[c]	.	.	.	.	.	.
[Smoking =2]	-1.674E-14	1.4151	.000	681	1.000	-2.779	2.779
[Smoking =3]	0[c]	.	.	.	.	.	.
[Average income=1]	7.028	2.3657	2.971	681	.003	2.383	11.673
[Average income=2]	7.042	2.3972	2.938	681	.003	2.336	11.749
[Average income=3]	0[c]	.	.	.	.	.	.
[Disease severity =1]	-26.280	2.3463	-11.200	681	.000	-30.887	-21.673
[Disease severity =2]	-18.624	1.7771	-10.480	681	.000	-22.114	-15.135
[Disease severity =3]	-26.665	2.2127	-12.051	681	.000	-31.010	-22.321

Parameter Estimates (q=0.5)[a,b]

Parameter	Coefficient	Std. Error	t	df	Sig.	95% Confidence Interval Lower Bound	Upper Bound
[Disease severity =4]	0[c]	.	.	.	.	.	.
[binary_chronic=.00]	3.107	2.5166	1.235	681	.217	-1.834	8.048
[binary_chronic=1.00]	0[c]	.	.	.	.	.	.

a. Dependent Variable: SGRQ_Sympt_Perc

b. Model: (Intercept), How many medications do you generally take?, Age, Disease duration, Gender , Educational level , Material status , Living in , Steroids inhaler, LAMA, SABA, Adherance_level, LABA, Smoking , Average income, Disease severity , binary_chronic

c. Set to zero because this parameter is redundant.

Prediction: How many medications do you generally take?

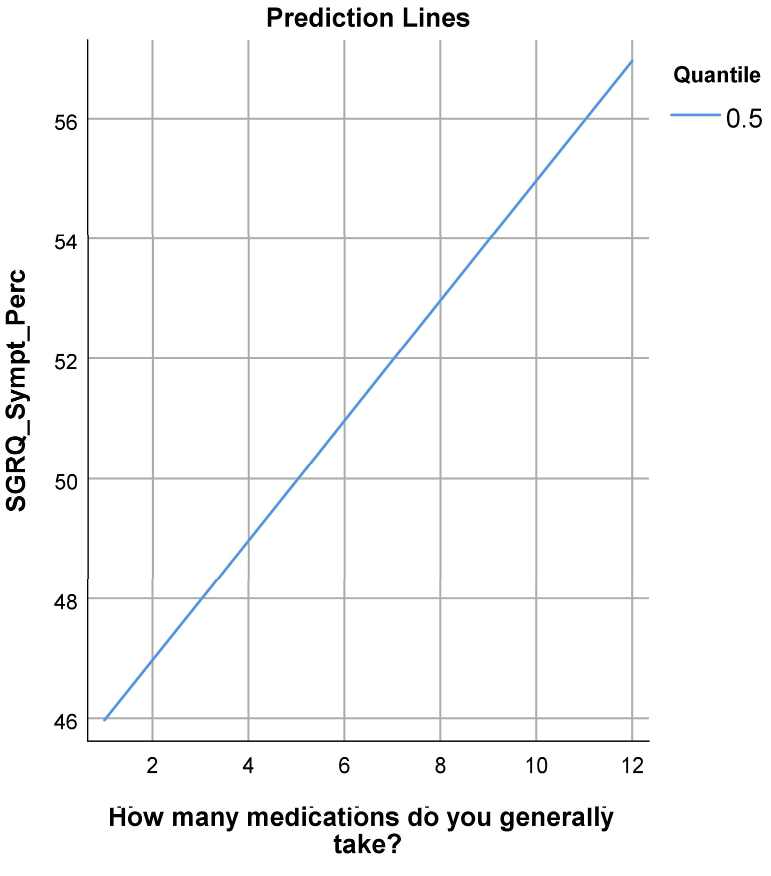

Prediction: Age

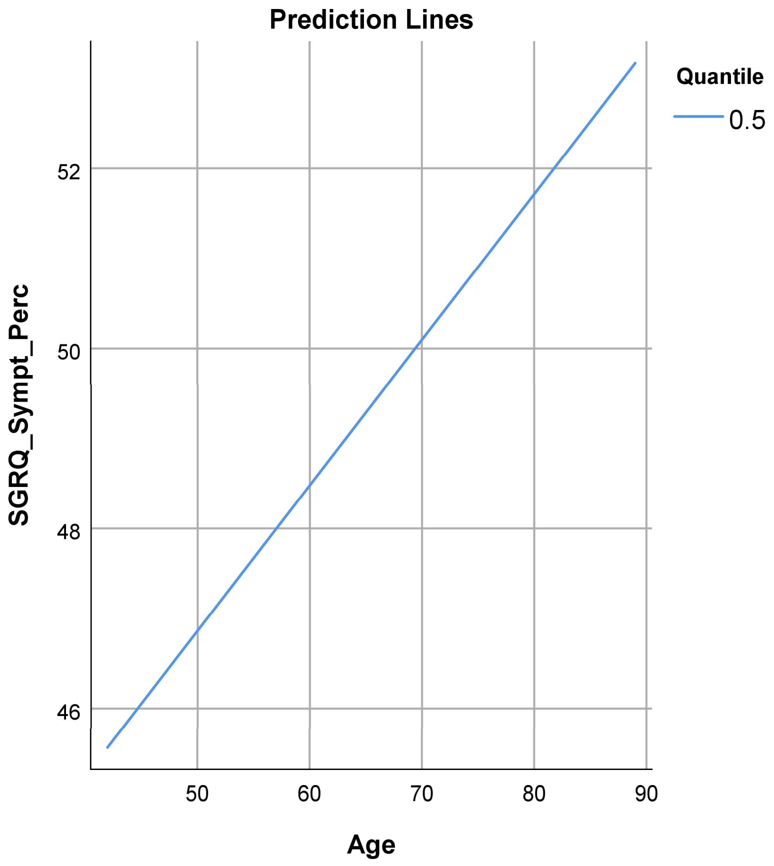

Prediction: Disease duration

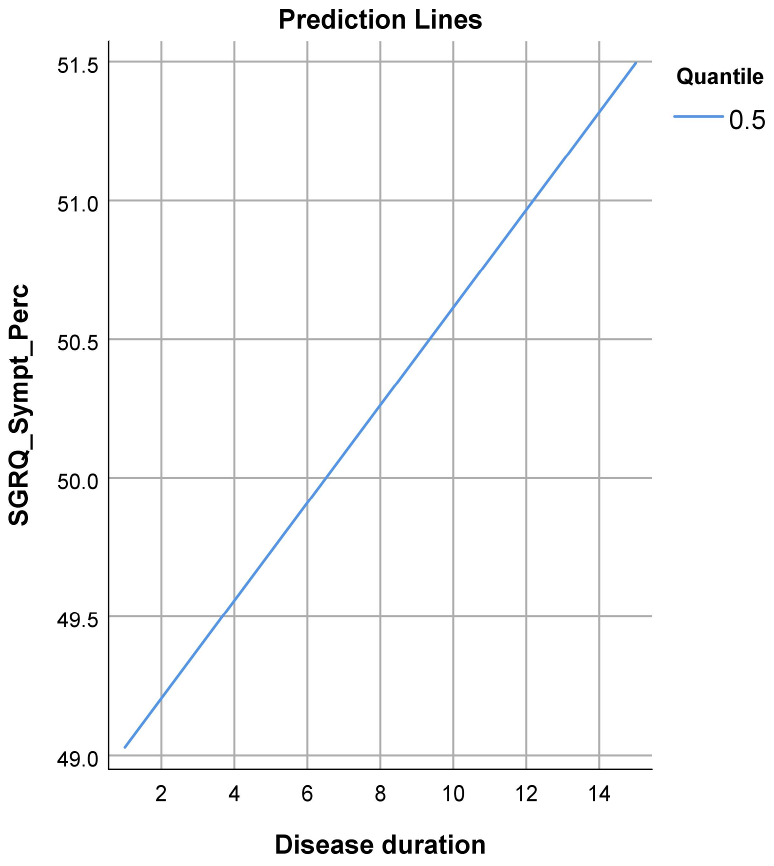

Prediction: Gender

Prediction Table[a,b,c]

Gender	q=0.5
Male	49.584
Female	47.954

a. Dependent Variable: SGRQ_Sympt_Perc

b. Model: (Intercept), How many medications do you generally take?, Age, Disease duration, Gender , Educational level , Material status , Living in , Steroids inhaler, LAMA, SABA, Adherance_level, LABA, Smoking , Average income, Disease severity , binary_chronic

c. Predictors in the model are evaluated at Educational level =Low,Material status =Married,Living in =Urban area,Steroids inhaler=Yes,LAMA=No,SABA=No,Adherance_level=non adhered,LABA=Yes, Smoking =Smoker,Average income=less than 600,Disease severity =B,binary_chronic=Yes,How many medications do you generally take?=4.62,Age=66.8262,Disease duration=4.1496

Prediction: Educational level

Prediction Table[a,b,c]

Educational level	q=0.5
Low	49.584
Moderate	51.861
High	47.745

a. Dependent Variable: SGRQ_Sympt_Perc

b. Model: (Intercept), How many medications do you generally take?, Age, Disease duration, Gender , Educational level , Material status , Living in , Steroids inhaler, LAMA, SABA, Adherance_level, LABA, Smoking , Average income, Disease severity , binary_chronic

c. Predictors in the model are evaluated at Gender =Male,Material status =Married,Living in =Urban area, Steroids inhaler=Yes,LAMA=No,SABA=No,Adherance_level=non adhered,LABA=Yes,Smoking =Smoker,Average income=less than 600,Disease severity =B,binary_chronic=Yes,How many medications do you generally take?=4.62,Age=66.8262,Disease duration=4.1496

Prediction: Material status

Prediction Table[a,b,c]

Material status	q=0.5
Married	49.584
Other	51.067

a. Dependent Variable: SGRQ_Sympt_Perc

b. Model: (Intercept), How many medications do you generally take?, Age, Disease duration, Gender , Educational level , Material status , Living in , Steroids inhaler, LAMA, SABA, Adherance_level, LABA, Smoking , Average income, Disease severity , binary_chronic

c. Predictors in the model are evaluated at Gender =Male,Educational level =Low,Living in =Urban area, Steroids inhaler=Yes,LAMA=No,SABA=No,Adherance_level=non adhered,LABA=Yes,Smoking =Smoker,Average income=less than 600,Disease severity =B,binary_chronic=Yes,How many medications do you generally take?=4.62,Age=66.8262,Disease duration=4.1496

Prediction: Living in

Prediction Table[a,b,c]

Living in	q=0.5
Rural area	60.311
Urban area	49.584

a. Dependent Variable: SGRQ_Sympt_Perc

b. Model: (Intercept), How many medications do you generally take?, Age, Disease duration, Gender , Educational level , Material status , Living in , Steroids inhaler, LAMA, SABA, Adherance_level, LABA, Smoking , Average income, Disease severity , binary_chronic

c. Predictors in the model are evaluated at Gender =Male,Educational level =Low,Material status =Married, Steroids inhaler=Yes,LAMA=No,SABA=No,Adherance_level=non adhered,LABA=Yes,Smoking =Smoker,Average income=less than 600,Disease severity =B,binary_chronic=Yes,How many medications do you generally take?=4.62,Age=66.8262,Disease duration=4.1496

Prediction: Steroids inhaler

Prediction Table[a,b,c]

Steroids inhaler	q=0.5
No	52.578
Yes	49.584

a. Dependent Variable: SGRQ_Sympt_Perc

b. Model: (Intercept), How many medications do you generally take?, Age, Disease duration, Gender , Educational level , Material status , Living in , Steroids inhaler, LAMA, SABA, Adherance_level, LABA, Smoking , Average income, Disease severity , binary_chronic

c. Predictors in the model are evaluated at Gender =Male,Educational level =Low,Material status =Married, Living in =Urban area,LAMA=No,SABA=No,Adherance_level=non adhered,LABA=Yes,Smoking =Smoker,Average income=less than 600,Disease severity =B,binary_chronic=Yes,How many medications do you generally take?=4.62,Age=66.8262,Disease duration=4.1496

Prediction: LAMA

Prediction Table[a,b,c]

LAMA	q=0.5
No	49.584
Yes	46.466

a. Dependent Variable: SGRQ_Sympt_Perc

b. Model: (Intercept), How many medications do you generally take?, Age, Disease duration, Gender , Educational level , Material status , Living in , Steroids inhaler, LAMA, SABA, Adherance_level, LABA, Smoking , Average income, Disease severity , binary_chronic

c. Predictors in the model are evaluated at Gender =Male,Educational level =Low,Material status =Married, Living in =Urban area,Steroids inhaler=Yes,SABA=No,Adherance_level=non adhered,LABA=Yes, Smoking =Smoker,Average income=less than 600,Disease severity =B,binary_chronic=Yes,How many medications do you generally take?=4.62,Age=66.8262,Disease duration=4.1496

Prediction: SABA

Prediction Table[a,b,c]

SABA	q=0.5
No	49.584
Yes	50.776

a. Dependent Variable: SGRQ_Sympt_Perc

b. Model: (Intercept), How many medications do you generally take?, Age, Disease duration, Gender , Educational level , Material status , Living in , Steroids inhaler, LAMA, SABA, Adherance_level, LABA, Smoking , Average income, Disease severity , binary_chronic

c. Predictors in the model are evaluated at Gender =Male,Educational level =Low,Material status =Married, Living in =Urban area,Steroids inhaler=Yes,LAMA=No,Adherance_level=non adhered,LABA=Yes, Smoking =Smoker,Average income=less than 600,Disease severity =B,binary_chronic=Yes,How many medications do you generally take?=4.62,Age=66.8262,Disease duration=4.1496

Prediction: Adherance_level

Prediction Table[a,b,c]

Adherance_level	q=0.5
adhered	41.827
non adhered	49.584

a. Dependent Variable: SGRQ_Sympt_Perc

b. Model: (Intercept), How many medications do you generally take?, Age, Disease duration, Gender , Educational level , Material status , Living in , Steroids inhaler, LAMA, SABA, Adherance_level, LABA, Smoking , Average income, Disease severity , binary_chronic

c. Predictors in the model are evaluated at Gender =Male,Educational level =Low,Material status =Married, Living in =Urban area,Steroids inhaler=Yes,LAMA=No,SABA=No,LABA=Yes,Smoking =Smoker,Average income=less than 600,Disease severity =B,binary_chronic=Yes,How many medications do you generally take?=4.62,Age=66.8262,Disease duration=4.1496

Prediction: LABA

Prediction Table[a,b,c]

LABA	q=0.5
No	42.556
Yes	49.584

a. Dependent Variable: SGRQ_Sympt_Perc

b. Model: (Intercept), How many medications do you generally take?, Age, Disease duration, Gender , Educational level , Material status , Living in , Steroids inhaler, LAMA, SABA, Adherance_level, LABA, Smoking , Average income, Disease severity , binary_chronic

c. Predictors in the model are evaluated at Gender =Male,Educational level =Low,Material status =Married, Living in =Urban area,Steroids inhaler=Yes,LAMA=No,SABA=No,Adherance_level=non adhered, Smoking =Smoker,Average income=less than 600,Disease severity =B,binary_chronic=Yes,How many medications do you generally take?=4.62,Age=66.8262,Disease duration=4.1496

Prediction: Smoking

Prediction Table[a,b,c]

Smoking	q=0.5
Former smoker	49.584
Smoker	49.584

a. Dependent Variable: SGRQ_Sympt_Perc

b. Model: (Intercept), How many medications do you generally take?, Age, Disease duration, Gender , Educational level , Material status , Living in , Steroids inhaler, LAMA, SABA, Adherance_level, LABA, Smoking , Average income, Disease severity , binary_chronic

c. Predictors in the model are evaluated at Gender =Male,Educational level =Low,Material status =Married, Living in =Urban area,Steroids inhaler=Yes,LAMA=No,SABA=No,Adherance_level=non adhered, LABA=Yes,Average income=less than 600,Disease severity =B,binary_chronic=Yes,How many medications do you generally take?=4.62,Age=66.8262,Disease duration=4.1496

Prediction: Average income

Prediction Table[a,b,c]

Average income	q=0.5
less than 600	49.584
600-1000	49.598
more than 1000	42.556

a. Dependent Variable: SGRQ_Sympt_Perc

b. Model: (Intercept), How many medications do you generally take?, Age, Disease duration, Gender , Educational level , Material status , Living in , Steroids inhaler, LAMA, SABA, Adherance_level, LABA, Smoking , Average income, Disease severity , binary_chronic

c. Predictors in the model are evaluated at Gender =Male,Educational level =Low,Material status =Married, Living in =Urban area,Steroids inhaler=Yes,LAMA=No,SABA=No,Adherance_level=non adhered, LABA=Yes,Smoking =Smoker,Disease severity =B,binary_chronic=Yes,How many medications do you generally take?=4.62,Age=66.8262,Disease duration=4.1496

Prediction: Disease severity

Prediction Table[a,b,c]

Disease severity	q=0.5
A	41.928
B	49.584
C	41.543
D	68.208

a. Dependent Variable: SGRQ_Sympt_Perc

b. Model: (Intercept), How many medications do you generally take?, Age, Disease duration, Gender , Educational level , Material status , Living in , Steroids inhaler, LAMA, SABA, Adherance_level, LABA, Smoking , Average income, Disease severity , binary_chronic

c. Predictors in the model are evaluated at Gender =Male,Educational level =Low,Material status =Married, Living in =Urban area,Steroids inhaler=Yes,LAMA=No,SABA=No,Adherance_level=non adhered, LABA=Yes,Smoking =Smoker,Average income=less than 600,binary_chronic=Yes,How many medications do you generally take?=4.62,Age=66.8262,Disease duration=4.1496

Prediction: binary_chronic

Prediction Table[a,b,c]

binary_chronic	q=0.5
No	52.690
Yes	49.584

a. Dependent Variable: SGRQ_Sympt_Perc

b. Model: (Intercept), How many medications do you generally take?, Age, Disease duration, Gender , Educational level , Material status , Living in , Steroids inhaler, LAMA, SABA, Adherance_level, LABA, Smoking , Average income, Disease severity , binary_chronic

c. Predictors in the model are evaluated at Gender =Male,Educational level =Low,Material status =Married, Living in =Urban area,Steroids inhaler=Yes,LAMA=No,SABA=No,Adherance_level=non adhered, LABA=Yes,Smoking =Smoker,Average income=less than 600,Disease severity =B,How many medications do you generally take?=4.62,Age=66.8262,Disease duration=4.1496

```
QUANTILE REGRESSION SGRQ_Activity_perc BY Sex Educational_level Marital_Status
 Residency
    Steroids_inhaler LAMA SABA Adherance_level LABA Smoking_status Income Dise
ase_severity
    binary_chronic WITH Number_of_medications_ingeneral Age Disease_duration
  /CRITERIA QUANTILE=0.5 METHOD=AUTO IID=TRUE BANDWIDTH=BOFINGER TOL=0.0000000
00001 CONV=0.000001
    MAXITER=2000 CILEVEL=95
  /MISSING CLASSMISSING=EXCLUDE
  /MODEL INTERCEPT=TRUE
  /PRINT PARAMETER
  /PLOT  PREDICTED_BY_OBSERVED=FALSE MAX_CATEGORIES=20
  /PREDICT_EFFECTS NUM_TOP_EFFECTS=20
  /SAVE  PRED.
```

Quantile Regression

Model Quality (q=0.5)[a,b,c]

Pseudo R Squared	.330
Mean Absolute Error (MAE)	13.4191

a. Dependent Variable: SGRQ_Activity_perc

b. Model: (Intercept), How many medications do you generally take?, Age, Disease duration, Gender , Educational level , Material status , Living in , Steroids inhaler, LAMA, SABA, Adherance_level, LABA, Smoking , Average income, Disease severity , binary_chronic

c. Method: Simplex algorithm

Parameter Estimates (q=0.5)[a,b]

Parameter	Coefficient	Std. Error	t	df	Sig.	95% Confidence Interval Lower Bound	95% Confidence Interval Upper Bound
(Intercept)	8.550	5.0895	1.680	681	.093	-1.443	18.543
How many medications do you generally take?	1.723	.3114	5.534	681	.000	1.112	2.335
Age	.578	.0481	12.034	681	.000	.484	.673
Disease duration	-.084	.1837	-.455	681	.649	-.444	.277
[Gender =0]	6.462	1.4696	4.397	681	.000	3.576	9.347
[Gender =1]	0[c]	.	.	.	.	.	.
[Educational level =1]	13.511	1.5243	8.864	681	.000	10.518	16.504
[Educational level =2]	.388	1.4449	.269	681	.788	-2.449	3.225
[Educational level =3]	0[c]	.	.	.	.	.	.
[Material status =2]	-7.842	2.0275	-3.868	681	.000	-11.822	-3.861
[Material status =3]	0[c]	.	.	.	.	.	.
[Living in =1]	-3.655	1.7040	-2.145	681	.032	-7.001	-.309
[Living in =2]	0[c]	.	.	.	.	.	.
[Steroids inhaler=.00]	-.121	1.1774	-.103	681	.918	-2.433	2.190
[Steroids inhaler=1.00]	0[c]	.	.	.	.	.	.
[LAMA=.00]	-2.161	1.2022	-1.798	681	.073	-4.521	.199
[LAMA=1.00]	0[c]	.	.	.	.	.	.
[SABA=.00]	4.167	1.4447	2.885	681	.004	1.331	7.004
[SABA=1.00]	0[c]	.	.	.	.	.	.
[Adherance_level=.00]	-2.896	1.2971	-2.233	681	.026	-5.443	-.350
[Adherance_level=1.00]	0[c]	.	.	.	.	.	.
[LABA=.00]	-13.692	1.1752	-11.651	681	.000	-16.000	-11.385
[LABA=1.00]	0[c]	.	.	.	.	.	.
[Smoking =2]	-.099	1.1271	-.088	681	.930	-2.312	2.114
[Smoking =3]	0[c]	.	.	.	.	.	.
[Average income=1]	-1.628	1.8843	-.864	681	.388	-5.327	2.072
[Average income=2]	-1.163	1.9093	-.609	681	.543	-4.911	2.586
[Average income=3]	0[c]	.	.	.	.	.	.
[Disease severity =1]	-16.035	1.8688	-8.580	681	.000	-19.704	-12.366
[Disease severity =2]	-4.377	1.4155	-3.092	681	.002	-7.156	-1.598
[Disease severity =3]	-5.229	1.7623	-2.967	681	.003	-8.689	-1.769

Parameter Estimates (q=0.5)[a,b]

Parameter	Coefficient	Std. Error	t	df	Sig.	95% Confidence Interval Lower Bound	95% Confidence Interval Upper Bound
[Disease severity =4]	0[c]	.	.	.	.	.	.
[binary_chronic=.00]	-19.519	2.0045	-9.738	681	.000	-23.454	-15.583
[binary_chronic=1.00]	0[c]	.	.	.	.	.	.

a. Dependent Variable: SGRQ_Activity_perc

b. Model: (Intercept), How many medications do you generally take?, Age, Disease duration, Gender , Educational level , Material status , Living in , Steroids inhaler, LAMA, SABA, Adherance_level, LABA, Smoking , Average income, Disease severity , binary_chronic

c. Set to zero because this parameter is redundant.

Prediction: How many medications do you generally take?

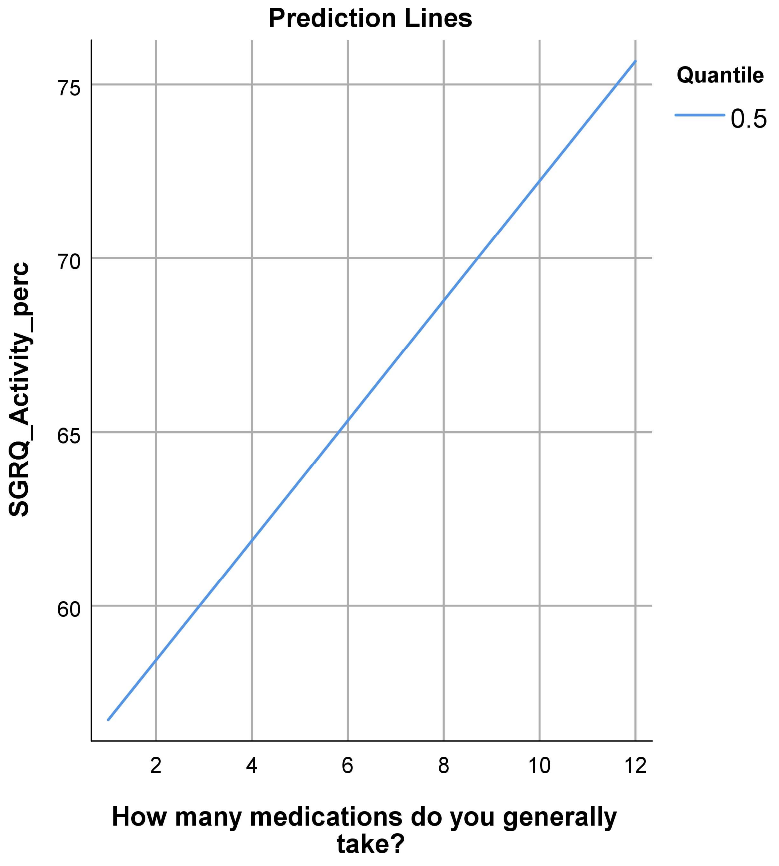

Prediction: Age

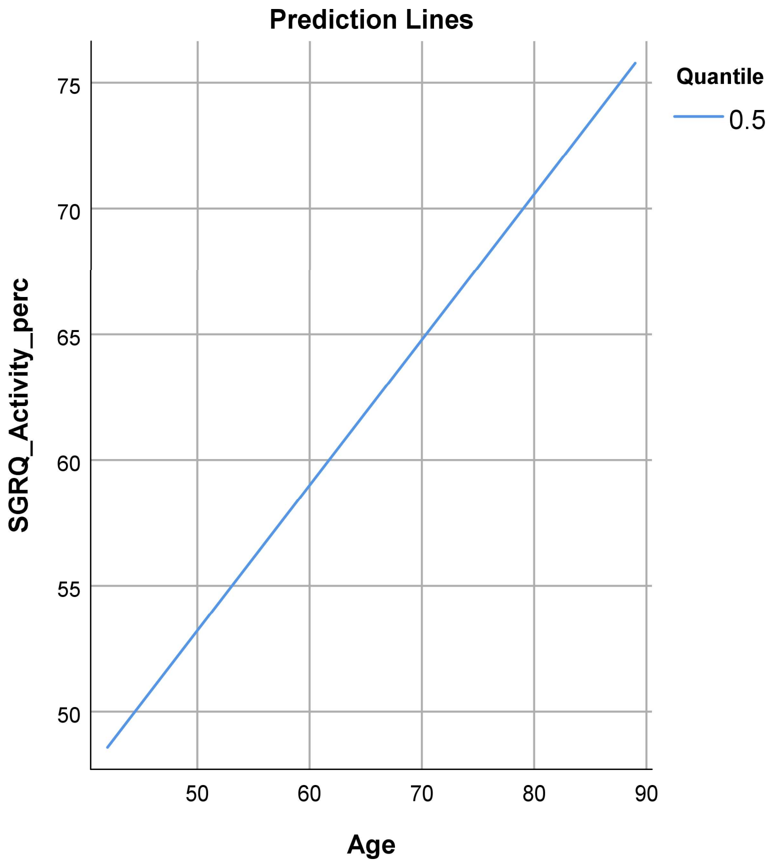

Prediction: Disease duration

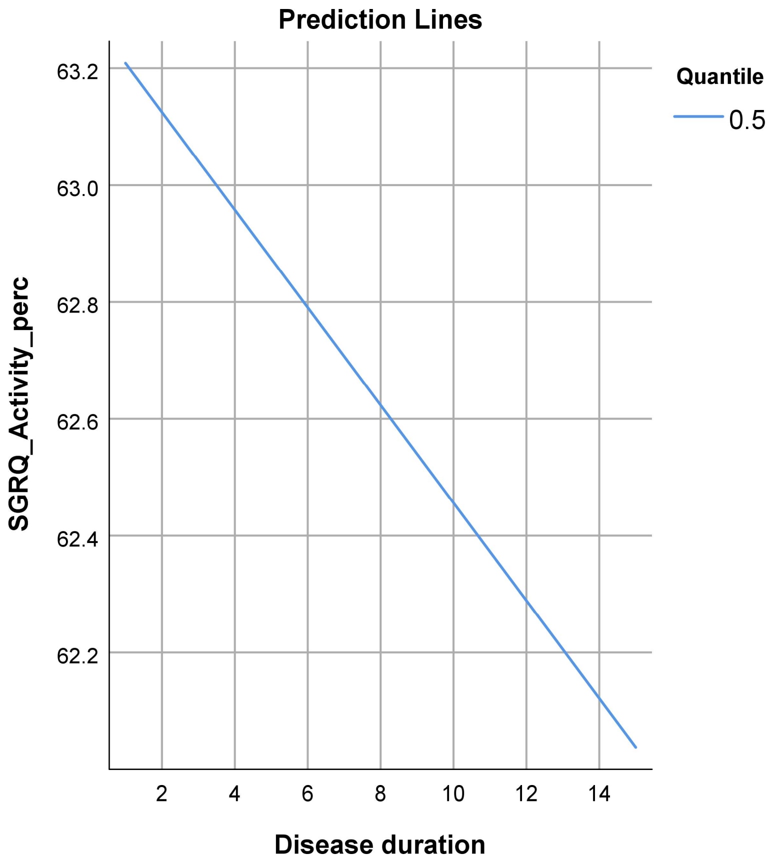

Prediction: Gender

Prediction Table[a,b,c]

Gender	q=0.5
Male	62.945
Female	56.484

a. Dependent Variable: SGRQ_Activity_perc

b. Model: (Intercept), How many medications do you generally take?, Age, Disease duration, Gender , Educational level , Material status , Living in , Steroids inhaler, LAMA, SABA, Adherance_level, LABA, Smoking , Average income, Disease severity , binary_chronic

c. Predictors in the model are evaluated at Educational level =Low,Material status =Married,Living in =Urban area,Steroids inhaler=Yes,LAMA=No,SABA=No,Adherance_level=non adhered,LABA=Yes, Smoking =Smoker,Average income=less than 600,Disease severity =B,binary_chronic=Yes,How many medications do you generally take?=4.62,Age=66.8262,Disease duration=4.1496

Prediction: Educational level

Prediction Table[a,b,c]

Educational level	q=0.5
Low	62.945
Moderate	49.822
High	49.434

a. Dependent Variable: SGRQ_Activity_perc

b. Model: (Intercept), How many medications do you generally take?, Age, Disease duration, Gender , Educational level , Material status , Living in , Steroids inhaler, LAMA, SABA, Adherance_level, LABA, Smoking , Average income, Disease severity , binary_chronic

c. Predictors in the model are evaluated at Gender =Male,Material status =Married,Living in =Urban area, Steroids inhaler=Yes,LAMA=No,SABA=No,Adherance_level=non adhered,LABA=Yes,Smoking =Smoker,Average income=less than 600,Disease severity =B,binary_chronic=Yes,How many medications do you generally take?=4.62,Age=66.8262,Disease duration=4.1496

Prediction: Material status

Prediction Table[a,b,c]

Material status	q=0.5
Married	62.945
Other	70.787

a. Dependent Variable: SGRQ_Activity_perc

b. Model: (Intercept), How many medications do you generally take?, Age, Disease duration, Gender , Educational level , Material status , Living in , Steroids inhaler, LAMA, SABA, Adherance_level, LABA, Smoking , Average income, Disease severity , binary_chronic

c. Predictors in the model are evaluated at Gender =Male,Educational level =Low,Living in =Urban area, Steroids inhaler=Yes,LAMA=No,SABA=No,Adherance_level=non adhered,LABA=Yes,Smoking =Smoker,Average income=less than 600,Disease severity =B,binary_chronic=Yes,How many medications do you generally take?=4.62,Age=66.8262,Disease duration=4.1496

Prediction: Living in

Prediction Table[a,b,c]

Living in	q=0.5
Rural area	59.290
Urban area	62.945

a. Dependent Variable: SGRQ_Activity_perc

b. Model: (Intercept), How many medications do you generally take?, Age, Disease duration, Gender , Educational level , Material status , Living in , Steroids inhaler, LAMA, SABA, Adherance_level, LABA, Smoking , Average income, Disease severity , binary_chronic

c. Predictors in the model are evaluated at Gender =Male,Educational level =Low,Material status =Married, Steroids inhaler=Yes,LAMA=No,SABA=No,Adherance_level=non adhered,LABA=Yes,Smoking =Smoker,Average income=less than 600,Disease severity =B,binary_chronic=Yes,How many medications do you generally take?=4.62,Age=66.8262,Disease duration=4.1496

Prediction: Steroids inhaler

Prediction Table[a,b,c]

Steroids inhaler	q=0.5
No	62.824
Yes	62.945

a. Dependent Variable: SGRQ_Activity_perc

b. Model: (Intercept), How many medications do you generally take?, Age, Disease duration, Gender , Educational level , Material status , Living in , Steroids inhaler, LAMA, SABA, Adherance_level, LABA, Smoking , Average income, Disease severity , binary_chronic

c. Predictors in the model are evaluated at Gender =Male,Educational level =Low,Material status =Married, Living in =Urban area,LAMA=No,SABA=No,Adherance_level=non adhered,LABA=Yes,Smoking =Smoker,Average income=less than 600,Disease severity =B,binary_chronic=Yes,How many medications do you generally take?=4.62,Age=66.8262,Disease duration=4.1496

Prediction: LAMA

Prediction Table[a,b,c]

LAMA	q=0.5
No	62.945
Yes	65.106

a. Dependent Variable: SGRQ_Activity_perc

b. Model: (Intercept), How many medications do you generally take?, Age, Disease duration, Gender , Educational level , Material status , Living in , Steroids inhaler, LAMA, SABA, Adherance_level, LABA, Smoking , Average income, Disease severity , binary_chronic

c. Predictors in the model are evaluated at Gender =Male,Educational level =Low,Material status =Married, Living in =Urban area,Steroids inhaler=Yes,SABA=No,Adherance_level=non adhered,LABA=Yes, Smoking =Smoker,Average income=less than 600,Disease severity =B,binary_chronic=Yes,How many medications do you generally take?=4.62,Age=66.8262,Disease duration=4.1496

Prediction: SABA

Prediction Table[a,b,c]

SABA	q=0.5
No	62.945
Yes	58.778

a. Dependent Variable: SGRQ_Activity_perc

b. Model: (Intercept), How many medications do you generally take?, Age, Disease duration, Gender , Educational level , Material status , Living in , Steroids inhaler, LAMA, SABA, Adherance_level, LABA, Smoking , Average income, Disease severity , binary_chronic

c. Predictors in the model are evaluated at Gender =Male,Educational level =Low,Material status =Married, Living in =Urban area,Steroids inhaler=Yes,LAMA=No,Adherance_level=non adhered,LABA=Yes, Smoking =Smoker,Average income=less than 600,Disease severity =B,binary_chronic=Yes,How many medications do you generally take?=4.62,Age=66.8262,Disease duration=4.1496

Prediction: Adherance_level

Prediction Table[a,b,c]

Adherance_level	q=0.5
adhered	60.049
non adhered	62.945

a. Dependent Variable: SGRQ_Activity_perc

b. Model: (Intercept), How many medications do you generally take?, Age, Disease duration, Gender , Educational level , Material status , Living in , Steroids inhaler, LAMA, SABA, Adherance_level, LABA, Smoking , Average income, Disease severity , binary_chronic

c. Predictors in the model are evaluated at Gender =Male,Educational level =Low,Material status =Married, Living in =Urban area,Steroids inhaler=Yes,LAMA=No,SABA=No,LABA=Yes,Smoking =Smoker,Average income=less than 600,Disease severity =B,binary_chronic=Yes,How many medications do you generally take?=4.62,Age=66.8262,Disease duration=4.1496

Prediction: LABA

Prediction Table[a,b,c]

LABA	q=0.5
No	49.253
Yes	62.945

a. Dependent Variable: SGRQ_Activity_perc

b. Model: (Intercept), How many medications do you generally take?, Age, Disease duration, Gender , Educational level , Material status , Living in , Steroids inhaler, LAMA, SABA, Adherance_level, LABA, Smoking , Average income, Disease severity , binary_chronic

c. Predictors in the model are evaluated at Gender =Male,Educational level =Low,Material status =Married, Living in =Urban area,Steroids inhaler=Yes,LAMA=No,SABA=No,Adherance_level=non adhered, Smoking =Smoker,Average income=less than 600,Disease severity =B,binary_chronic=Yes,How many medications do you generally take?=4.62,Age=66.8262,Disease duration=4.1496

Prediction: Smoking

Prediction Table[a,b,c]

Smoking	q=0.5
Former smoker	62.846
Smoker	62.945

a. Dependent Variable: SGRQ_Activity_perc

b. Model: (Intercept), How many medications do you generally take?, Age, Disease duration, Gender , Educational level , Material status , Living in , Steroids inhaler, LAMA, SABA, Adherance_level, LABA, Smoking , Average income, Disease severity , binary_chronic

c. Predictors in the model are evaluated at Gender =Male,Educational level =Low,Material status =Married, Living in =Urban area,Steroids inhaler=Yes,LAMA=No,SABA=No,Adherance_level=non adhered, LABA=Yes,Average income=less than 600,Disease severity =B,binary_chronic=Yes,How many medications do you generally take?=4.62,Age=66.8262,Disease duration=4.1496

Prediction: Average income

Prediction Table[a,b,c]

Average income	q=0.5
less than 600	62.945
600-1000	63.410
more than 1000	64.573

a. Dependent Variable: SGRQ_Activity_perc

b. Model: (Intercept), How many medications do you generally take?, Age, Disease duration, Gender , Educational level , Material status , Living in , Steroids inhaler, LAMA, SABA, Adherance_level, LABA, Smoking , Average income, Disease severity , binary_chronic

c. Predictors in the model are evaluated at Gender =Male,Educational level =Low,Material status =Married, Living in =Urban area,Steroids inhaler=Yes,LAMA=No,SABA=No,Adherance_level=non adhered, LABA=Yes,Smoking =Smoker,Disease severity =B,binary_chronic=Yes,How many medications do you generally take?=4.62,Age=66.8262,Disease duration=4.1496

Prediction: Disease severity

Prediction Table[a,b,c]

Disease severity	q=0.5
A	51.287
B	62.945
C	62.093
D	67.322

a. Dependent Variable: SGRQ_Activity_perc

b. Model: (Intercept), How many medications do you generally take?, Age, Disease duration, Gender , Educational level , Material status , Living in , Steroids inhaler, LAMA, SABA, Adherance_level, LABA, Smoking , Average income, Disease severity , binary_chronic

c. Predictors in the model are evaluated at Gender =Male,Educational level =Low,Material status =Married, Living in =Urban area,Steroids inhaler=Yes,LAMA=No,SABA=No,Adherance_level=non adhered, LABA=Yes,Smoking =Smoker,Average income=less than 600,binary_chronic=Yes,How many medications do you generally take?=4.62,Age=66.8262,Disease duration=4.1496

Prediction: binary_chronic

Prediction Table[a,b,c]

binary_chronic	q=0.5
No	43.426
Yes	62.945

a. Dependent Variable: SGRQ_Activity_perc

b. Model: (Intercept), How many medications do you generally take?, Age, Disease duration, Gender , Educational level , Material status , Living in , Steroids inhaler, LAMA, SABA, Adherance_level, LABA, Smoking , Average income, Disease severity , binary_chronic

c. Predictors in the model are evaluated at Gender =Male,Educational level =Low,Material status =Married, Living in =Urban area,Steroids inhaler=Yes,LAMA=No,SABA=No,Adherance_level=non adhered, LABA=Yes,Smoking =Smoker,Average income=less than 600,Disease severity =B,How many medications do you generally take?=4.62,Age=66.8262,Disease duration=4.1496

```
QUANTILE REGRESSION SGRQ_Impact_per BY Sex Educational_level Marital_Status Re
sidency
    Steroids_inhaler LAMA SABA Adherance_level LABA Smoking_status Income Dise
ase_severity
    binary_chronic WITH Number_of_medications_ingeneral Age Disease_duration
  /CRITERIA QUANTILE=0.5 METHOD=AUTO IID=TRUE BANDWIDTH=BOFINGER TOL=0.0000000
00001 CONV=0.000001
    MAXITER=2000 CILEVEL=95
  /MISSING CLASSMISSING=EXCLUDE
  /MODEL INTERCEPT=TRUE
  /PRINT PARAMETER
  /PLOT  PREDICTED_BY_OBSERVED=FALSE MAX_CATEGORIES=20
  /PREDICT_EFFECTS NUM_TOP_EFFECTS=20
  /SAVE  PRED.
```

Quantile Regression

Model Quality (q=0.5)[a,b,c]

Pseudo R Squared	.333
Mean Absolute Error (MAE)	16.3052

a. Dependent Variable: SGRQ_Impact_per

b. Model: (Intercept), How many medications do you generally take?, Age, Disease duration, Gender , Educational level , Material status , Living in , Steroids inhaler, LAMA, SABA, Adherance_level, LABA, Smoking , Average income, Disease severity , binary_chronic

c. Method: Simplex algorithm

Parameter Estimates (q=0.5)[a,b]

Parameter	Coefficient	Std. Error	t	df	Sig.	95% Confidence Interval Lower Bound	95% Confidence Interval Upper Bound
(Intercept)	50.861	9.1343	5.568	681	.000	32.926	68.795
How many medications do you generally take?	2.057	.5588	3.681	681	.000	.960	3.155
Age	.478	.0863	5.537	681	.000	.308	.647
Disease duration	-.017	.3297	-.051	681	.960	-.664	.631
[Gender =0]	11.630	2.6375	4.409	681	.000	6.451	16.808
[Gender =1]	0[c]	.	.	.	.	.	.
[Educational level =1]	8.864	2.7358	3.240	681	.001	3.493	14.236
[Educational level =2]	1.351	2.5933	.521	681	.603	-3.741	6.443
[Educational level =3]	0[c]	.	.	.	.	.	.
[Material status =2]	-21.346	3.6388	-5.866	681	.000	-28.490	-14.201
[Material status =3]	0[c]	.	.	.	.	.	.
[Living in =1]	-11.458	3.0582	-3.747	681	.000	-17.463	-5.453
[Living in =2]	0[c]	.	.	.	.	.	.
[Steroids inhaler=.00]	-6.041	2.1130	-2.859	681	.004	-10.189	-1.892
[Steroids inhaler=1.00]	0[c]	.	.	.	.	.	.
[LAMA=.00]	-18.173	2.1576	-8.423	681	.000	-22.410	-13.937
[LAMA=1.00]	0[c]	.	.	.	.	.	.
[SABA=.00]	-2.000	2.5928	-.771	681	.441	-7.091	3.091
[SABA=1.00]	0[c]	.	.	.	.	.	.
[Adherance_level=.00]	-10.413	2.3279	-4.473	681	.000	-14.983	-5.842
[Adherance_level=1.00]	0[c]	.	.	.	.	.	.
[LABA=.00]	-5.826	2.1092	-2.762	681	.006	-9.967	-1.685
[LABA=1.00]	0[c]	.	.	.	.	.	.
[Smoking =2]	-3.779	2.0229	-1.868	681	.062	-7.751	.193
[Smoking =3]	0[c]	.	.	.	.	.	.
[Average income=1]	1.804	3.3818	.533	681	.594	-4.836	8.444
[Average income=2]	2.966	3.4268	.865	681	.387	-3.763	9.694
[Average income=3]	0[c]	.	.	.	.	.	.
[Disease severity =1]	-28.077	3.3541	-8.371	681	.000	-34.663	-21.491
[Disease severity =2]	-7.561	2.5404	-2.976	681	.003	-12.549	-2.573
[Disease severity =3]	-8.948	3.1630	-2.829	681	.005	-15.158	-2.737

Parameter Estimates (q=0.5)[a,b]

Parameter	Coefficient	Std. Error	t	df	Sig.	95% Confidence Interval Lower Bound	95% Confidence Interval Upper Bound
[Disease severity =4]	0[c]	.	.	.	.	.	.
[binary_chronic=.00]	-19.883	3.5975	-5.527	681	.000	-26.947	-12.820
[binary_chronic=1.00]	0[c]	.	.	.	.	.	.

a. Dependent Variable: SGRQ_Impact_per

b. Model: (Intercept), How many medications do you generally take?, Age, Disease duration, Gender , Educational level , Material status , Living in , Steroids inhaler, LAMA, SABA, Adherance_level, LABA, Smoking , Average income, Disease severity , binary_chronic

c. Set to zero because this parameter is redundant.

Prediction: How many medications do you generally take?

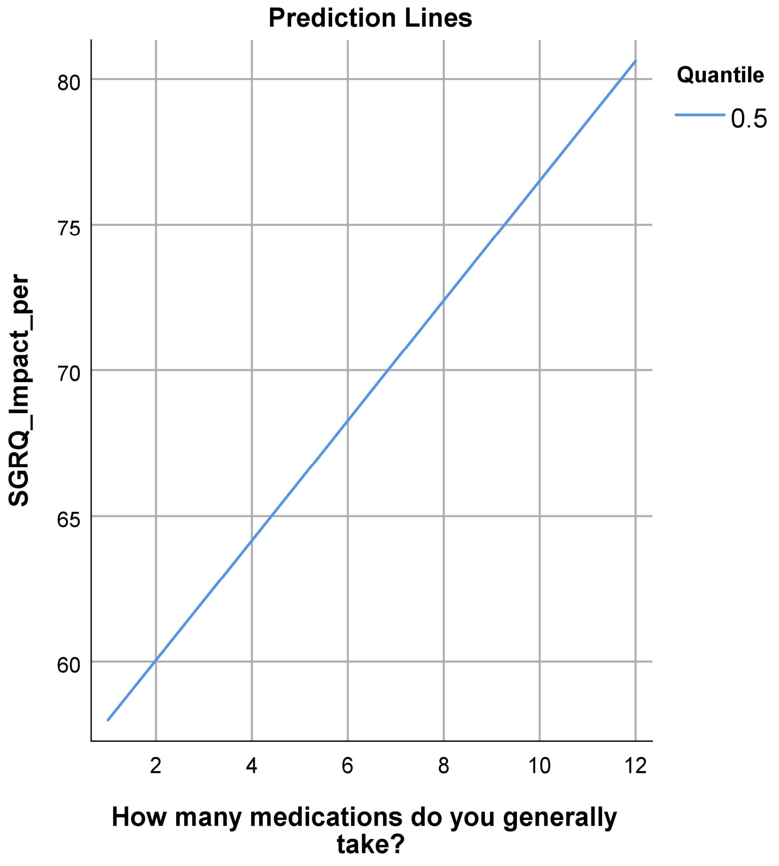

Prediction: Age

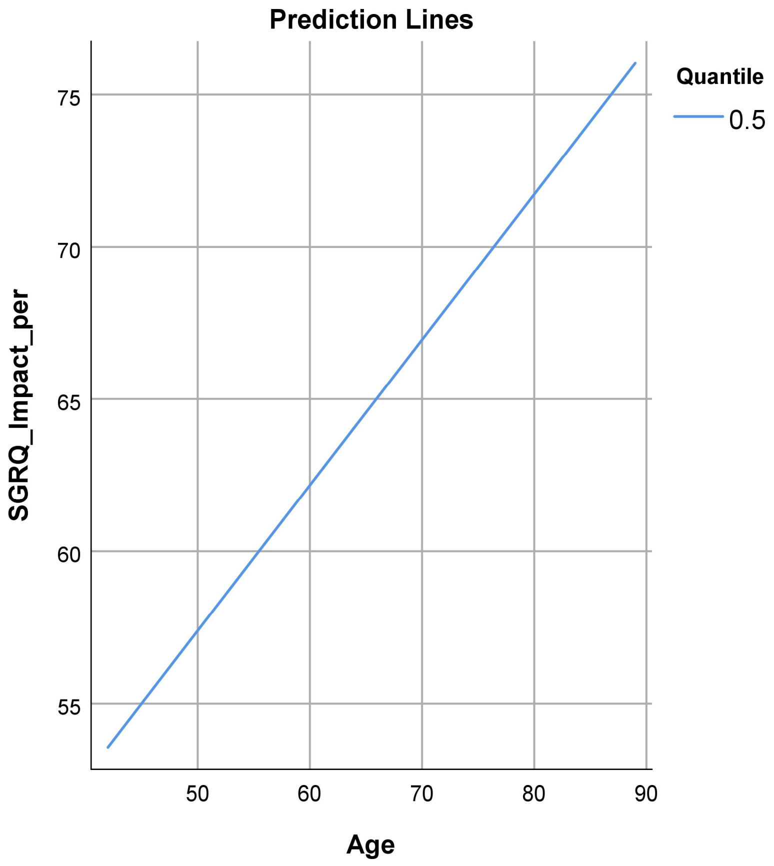

Prediction: Disease duration

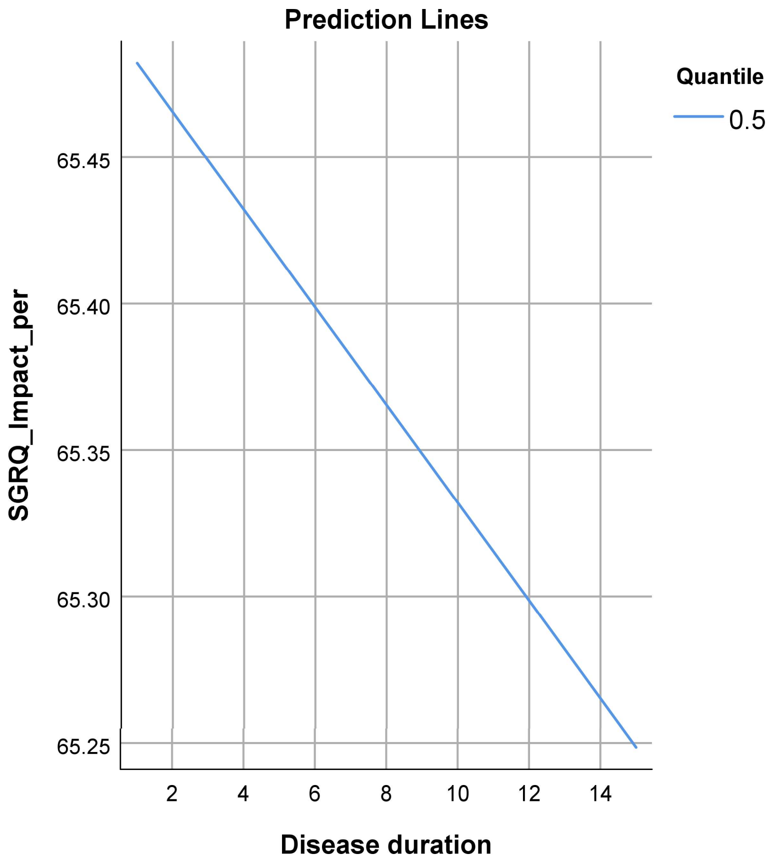

Prediction: Gender

Prediction Table[a,b,c]

Gender	q=0.5
Male	65.430
Female	53.800

a. Dependent Variable: SGRQ_Impact_per

b. Model: (Intercept), How many medications do you generally take?, Age, Disease duration, Gender , Educational level , Material status , Living in , Steroids inhaler, LAMA, SABA, Adherance_level, LABA, Smoking , Average income, Disease severity , binary_chronic

c. Predictors in the model are evaluated at Educational level =Low,Material status =Married,Living in =Urban area,Steroids inhaler=Yes,LAMA=No,SABA=No,Adherance_level=non adhered,LABA=Yes, Smoking =Smoker,Average income=less than 600,Disease severity =B,binary_chronic=Yes,How many medications do you generally take?=4.62,Age=66.8262,Disease duration=4.1496

Prediction: Educational level

Prediction Table[a,b,c]

Educational level	q=0.5
Low	65.430
Moderate	57.916
High	56.565

a. Dependent Variable: SGRQ_Impact_per

b. Model: (Intercept), How many medications do you generally take?, Age, Disease duration, Gender , Educational level , Material status , Living in , Steroids inhaler, LAMA, SABA, Adherance_level, LABA, Smoking , Average income, Disease severity , binary_chronic

c. Predictors in the model are evaluated at Gender =Male,Material status =Married,Living in =Urban area, Steroids inhaler=Yes,LAMA=No,SABA=No,Adherance_level=non adhered,LABA=Yes,Smoking =Smoker,Average income=less than 600,Disease severity =B,binary_chronic=Yes,How many medications do you generally take?=4.62,Age=66.8262,Disease duration=4.1496

Prediction: Material status

Prediction Table[a,b,c]

Material status	q=0.5
Married	65.430
Other	86.775

a. Dependent Variable: SGRQ_Impact_per

b. Model: (Intercept), How many medications do you generally take?, Age, Disease duration, Gender , Educational level , Material status , Living in , Steroids inhaler, LAMA, SABA, Adherance_level, LABA, Smoking , Average income, Disease severity , binary_chronic

c. Predictors in the model are evaluated at Gender =Male,Educational level =Low,Living in =Urban area, Steroids inhaler=Yes,LAMA=No,SABA=No,Adherance_level=non adhered,LABA=Yes,Smoking =Smoker,Average income=less than 600,Disease severity =B,binary_chronic=Yes,How many medications do you generally take?=4.62,Age=66.8262,Disease duration=4.1496

Prediction: Living in

Prediction Table[a,b,c]

Living in	q=0.5
Rural area	53.971
Urban area	65.430

a. Dependent Variable: SGRQ_Impact_per

b. Model: (Intercept), How many medications do you generally take?, Age, Disease duration, Gender , Educational level , Material status , Living in , Steroids inhaler, LAMA, SABA, Adherance_level, LABA, Smoking , Average income, Disease severity , binary_chronic

c. Predictors in the model are evaluated at Gender =Male,Educational level =Low,Material status =Married, Steroids inhaler=Yes,LAMA=No,SABA=No,Adherance_level=non adhered,LABA=Yes,Smoking =Smoker,Average income=less than 600,Disease severity =B,binary_chronic=Yes,How many medications do you generally take?=4.62,Age=66.8262,Disease duration=4.1496

Prediction: Steroids inhaler

Prediction Table[a,b,c]

Steroids inhaler	q=0.5
No	59.389
Yes	65.430

a. Dependent Variable: SGRQ_Impact_per

b. Model: (Intercept), How many medications do you generally take?, Age, Disease duration, Gender , Educational level , Material status , Living in , Steroids inhaler, LAMA, SABA, Adherance_level, LABA, Smoking , Average income, Disease severity , binary_chronic

c. Predictors in the model are evaluated at Gender =Male,Educational level =Low,Material status =Married, Living in =Urban area,LAMA=No,SABA=No,Adherance_level=non adhered,LABA=Yes,Smoking =Smoker,Average income=less than 600,Disease severity =B,binary_chronic=Yes,How many medications do you generally take?=4.62,Age=66.8262,Disease duration=4.1496

Prediction: LAMA

Prediction Table[a,b,c]

LAMA	q=0.5
No	65.430
Yes	83.603

a. Dependent Variable: SGRQ_Impact_per

b. Model: (Intercept), How many medications do you generally take?, Age, Disease duration, Gender , Educational level , Material status , Living in , Steroids inhaler, LAMA, SABA, Adherance_level, LABA, Smoking , Average income, Disease severity , binary_chronic

c. Predictors in the model are evaluated at Gender =Male,Educational level =Low,Material status =Married, Living in =Urban area,Steroids inhaler=Yes,SABA=No,Adherance_level=non adhered,LABA=Yes, Smoking =Smoker,Average income=less than 600,Disease severity =B,binary_chronic=Yes,How many medications do you generally take?=4.62,Age=66.8262,Disease duration=4.1496

Prediction: SABA

Prediction Table[a,b,c]

SABA	q=0.5
No	65.430
Yes	67.430

a. Dependent Variable: SGRQ_Impact_per

b. Model: (Intercept), How many medications do you generally take?, Age, Disease duration, Gender , Educational level , Material status , Living in , Steroids inhaler, LAMA, SABA, Adherance_level, LABA, Smoking , Average income, Disease severity , binary_chronic

c. Predictors in the model are evaluated at Gender =Male,Educational level =Low,Material status =Married, Living in =Urban area,Steroids inhaler=Yes,LAMA=No,Adherance_level=non adhered,LABA=Yes, Smoking =Smoker,Average income=less than 600,Disease severity =B,binary_chronic=Yes,How many medications do you generally take?=4.62,Age=66.8262,Disease duration=4.1496

Prediction: Adherance_level

Prediction Table[a,b,c]

Adherance_level	q=0.5
adhered	55.017
non adhered	65.430

a. Dependent Variable: SGRQ_Impact_per

b. Model: (Intercept), How many medications do you generally take?, Age, Disease duration, Gender , Educational level , Material status , Living in , Steroids inhaler, LAMA, SABA, Adherance_level, LABA, Smoking , Average income, Disease severity , binary_chronic

c. Predictors in the model are evaluated at Gender =Male,Educational level =Low,Material status =Married, Living in =Urban area,Steroids inhaler=Yes,LAMA=No,SABA=No,LABA=Yes,Smoking =Smoker,Average income=less than 600,Disease severity =B,binary_chronic=Yes,How many medications do you generally take?=4.62,Age=66.8262,Disease duration=4.1496

Prediction: LABA

Prediction Table[a,b,c]

LABA	q=0.5
No	59.604
Yes	65.430

a. Dependent Variable: SGRQ_Impact_per

b. Model: (Intercept), How many medications do you generally take?, Age, Disease duration, Gender , Educational level , Material status , Living in , Steroids inhaler, LAMA, SABA, Adherance_level, LABA, Smoking , Average income, Disease severity , binary_chronic

c. Predictors in the model are evaluated at Gender =Male,Educational level =Low,Material status =Married, Living in =Urban area,Steroids inhaler=Yes,LAMA=No,SABA=No,Adherance_level=non adhered, Smoking =Smoker,Average income=less than 600,Disease severity =B,binary_chronic=Yes,How many medications do you generally take?=4.62,Age=66.8262,Disease duration=4.1496

Prediction: Smoking

Prediction Table[a,b,c]

Smoking	q=0.5
Former smoker	61.650
Smoker	65.430

a. Dependent Variable: SGRQ_Impact_per

b. Model: (Intercept), How many medications do you generally take?, Age, Disease duration, Gender , Educational level , Material status , Living in , Steroids inhaler, LAMA, SABA, Adherance_level, LABA, Smoking , Average income, Disease severity , binary_chronic

c. Predictors in the model are evaluated at Gender =Male,Educational level =Low,Material status =Married, Living in =Urban area,Steroids inhaler=Yes,LAMA=No,SABA=No,Adherance_level=non adhered, LABA=Yes,Average income=less than 600,Disease severity =B,binary_chronic=Yes,How many medications do you generally take?=4.62,Age=66.8262,Disease duration=4.1496

Prediction: Average income

Prediction Table[a,b,c]

Average income	q=0.5
less than 600	65.430
600-1000	66.591
more than 1000	63.625

a. Dependent Variable: SGRQ_Impact_per

b. Model: (Intercept), How many medications do you generally take?, Age, Disease duration, Gender , Educational level , Material status , Living in , Steroids inhaler, LAMA, SABA, Adherance_level, LABA, Smoking , Average income, Disease severity , binary_chronic

c. Predictors in the model are evaluated at Gender =Male,Educational level =Low,Material status =Married, Living in =Urban area,Steroids inhaler=Yes,LAMA=No,SABA=No,Adherance_level=non adhered, LABA=Yes,Smoking =Smoker,Disease severity =B,binary_chronic=Yes,How many medications do you generally take?=4.62,Age=66.8262,Disease duration=4.1496

Prediction: Disease severity

Prediction Table[a,b,c]

Disease severity	q=0.5
A	44.914
B	65.430
C	64.043
D	72.991

a. Dependent Variable: SGRQ_Impact_per

b. Model: (Intercept), How many medications do you generally take?, Age, Disease duration, Gender , Educational level , Material status , Living in , Steroids inhaler, LAMA, SABA, Adherance_level, LABA, Smoking , Average income, Disease severity , binary_chronic

c. Predictors in the model are evaluated at Gender =Male,Educational level =Low,Material status =Married, Living in =Urban area,Steroids inhaler=Yes,LAMA=No,SABA=No,Adherance_level=non adhered, LABA=Yes,Smoking =Smoker,Average income=less than 600,binary_chronic=Yes,How many medications do you generally take?=4.62,Age=66.8262,Disease duration=4.1496

Prediction: binary_chronic

Prediction Table[a,b,c]

binary_chronic	q=0.5
No	45.546
Yes	65.430

a. Dependent Variable: SGRQ_Impact_per

b. Model: (Intercept), How many medications do you generally take?, Age, Disease duration, Gender , Educational level , Material status , Living in , Steroids inhaler, LAMA, SABA, Adherance_level, LABA, Smoking , Average income, Disease severity , binary_chronic

c. Predictors in the model are evaluated at Gender =Male,Educational level =Low,Material status =Married, Living in =Urban area,Steroids inhaler=Yes,LAMA=No,SABA=No,Adherance_level=non adhered, LABA=Yes,Smoking =Smoker,Average income=less than 600,Disease severity =B,How many medications do you generally take?=4.62,Age=66.8262,Disease duration=4.1496

Custom Tables

		Median	Percentile 25	Percentile 75	Count	Column N %
Age		68.00	58.00	77.00		
Gender	Male				552	78.6%
	Female				150	21.4%
Educational level	Low				252	35.9%
	Moderate				228	32.5%
	High				222	31.6%
Average income	less than 600				330	47.0%
	600-1000				290	41.3%
	more than 1000				82	11.7%
Material status	Not married				0	0.0%
	Married				642	91.5%
	Other				60	8.5%
Smoking	Former smoker				295	42.0%
	Smoker				407	58.0%
Living conditions	Alone				49	7.0%
	Not alone				653	93.0%
Living in	Rural area				92	13.1%
	Urban area				610	86.9%
Do you have pets?	No				636	90.6%
	Yes				66	9.4%

Custom Tables

		Median	Percentile 25	Percentile 75	Count	Column N %
Disease duration		4.00	2.00	5.00		
Hypertension	No				192	27.4%
	Yes				510	72.6%
Heart diseases	No				366	52.1%
	Yes				336	47.9%
Diabetes	No				348	49.6%
	Yes				354	50.4%
Number of COPD medications		2	2	2		
How many medications do you generally take?		5	3	6		
Steroids inhaler	No				222	31.6%
	Yes				480	68.4%
Oral steroids	No				678	96.6%
	Yes				24	3.4%
LABA	No				272	38.7%
	Yes				430	61.3%
LAMA	No				396	56.4%
	Yes				306	43.6%
SABA	No				564	80.3%
	Yes				138	19.7%
Do you use oxygen cylinders?	No				546	77.8%
	Yes				156	22.2%
Do you have concerns about the SE of COPD medication?	No				594	84.6%
	Yes				108	15.4%
Your evaluation for the drug effectiveness?	Not good				54	7.7%
	Good				414	59.0%
	Excellent				234	33.3%
Anxiety level	normal				414	59.0%
	abnormal				288	41.0%
Depression level	normal				318	45.3%
	abnormal				384	54.7%
Adherance_level	adhered				225	32.1%
	non adhered				477	67.9%
Disease severity	A				114	16.2%
	B				282	40.2%
	C				108	15.4%
	D				198	28.2%
SGRQ_Sympt_Perc		45.20	32.76	59.82		
SGRQ_Activity_perc		51.65	22.54	67.64		
SGRQ_Impact_per		58.74	29.02	76.30		
SGRQ_Perc		55.15	34.00	67.81		
